# Rethinking large-scale phylogenomics with EukPhylo v.1.0, a flexible toolkit to enable phylogeny-informed data curation and analyses of diverse eukaryotic lineages

**DOI:** 10.1128/mbio.01770-25

**Published:** 2025-08-27

**Authors:** Laura A. Katz, Marie Leleu, Godwin Ani, Rebecca Gawron, Auden Cote-L'Heureux

**Affiliations:** 1 Department of Biological Sciences, Smith College 6089, Northampton, Massachusetts, USA; 2 Program in Organismic Biology and Evolution, University of Massachusetts Amherst 14707, Amherst, Massachusetts, USA; McMaster University, Hamilton, Ontario, Canada

**Keywords:** phylogenomic standards, single-cell transcriptomes, phylogenetics, protists, eukaryotic tree of life, microbiome, contamination

## Abstract

Eukaryotic diversity is largely microbial, with macroscopic lineages (plants, animals, and fungi) nesting among a plethora of diverse protists. Our understanding of the evolutionary relationships among eukaryotes is rapidly advancing through ’omics analyses, but phylogenomic analyses are challenging for microeukaryotes, particularly uncultivable lineages, as single-cell sequencing approaches generate a mixture of sequences from hosts, associated microbiomes, and contaminants. Moreover, many analyses of eukaryotic gene families and phylogenies rely on boutique data sets and methods that are challenging for other research groups to replicate. To address these challenges, we present EukPhylo v.1.0, a modular, user-friendly pipeline that enables effective data curation through phylogeny-informed contamination removal, estimation of homologous gene families (GFs), and generation of both multisequence alignments and gene trees. For the GF assignment, we provide the “Hook Database” of ~15,000 ancient GFs, which users can easily replace with a set of gene families of interest. We demonstrate the power of EukPhylo, including a suite of stand-alone utilities, through phylogenomic analyses of 500 conserved GFs sampled from 1,000 diverse species of eukaryotes, bacteria, and archaea. We show improvements in estimates of the eukaryotic tree of life, recovering clades that are well established in the literature, through successive rounds of curation using the EukPhylo contamination loop. The final trees corroborate numerous hypotheses in the literature (e.g., Opisthokonta, Rhizaria, Amoebozoa) while challenging others (e.g., CRuMs, Obazoa, Diaphoretickes). The flexibility and transparency of EukPhylo set new standards for curation of ’omics data for future studies.

**IMPORTANCE:**

Illuminating the diversity of microbial lineages is essential for estimating the tree of life and characterizing principles of genome evolution. However, analyses of microbial eukaryotes (e.g., flagellates, amoebae) are complicated by both the paucity of reference genomes and the prevalence of contamination (e.g., by symbionts, microbiomes). EukPhylo v.1.0 enables taxon-rich analyses “on the fly” as users can choose optimal gene families for their focal taxa and then use replicable approaches to curate data in estimating both gene and species trees. With multiple entry points and curated data sets from up to 15,000 gene families from 1,000 taxa ready for use, EukPhylo provides a powerful launching point for researchers interested in the evolution of eukaryotes.

## INTRODUCTION

Most of our knowledge about the nature and evolution of eukaryotic life has emerged from studies of macroscopic organisms, with a focus on “model” lineages such as *Drosophila* and *Arabidopsis*. However, such models represent relatively narrow slices of the eukaryotic tree of life (EToL) as the bulk of eukaryotic diversity is microbial (e.g., references 1, 2). Insights from microbial eukaryotes (a.k.a. protists) expand our understanding of the “rules” of evolution by their tremendous diversity of morphologies, life cycles, and genome properties (3, 4). The gap in knowledge about microbial eukaryotes can be most efficiently filled through taxon-rich phylogenomic analysis methods. However, current practices often rely on boutique data sets and decisions (e.g., in removing contaminants and identifying orthologs) that lack independence and can be challenging to replicate (e.g., references 2, 5–7). To address these challenges, we developed EukPhylo v.1.0, a flexible phylogenomic pipeline designed for replicable analyses of diverse eukaryotes. EukPhylo includes curated data sets from diverse lineages, a workflow to process ‘omics data and to deploy phylogeny-informed contamination removal, and a suite of utilities to enable efficient estimation of gene families (GFs) and phylogenies.

Phylogenomic inference faces numerous challenges, including incongruence among loci, long-branch attraction (8, 9), and lateral gene transfer, which confound inferences (10). These issues are especially prevalent in microeukaryotes, where whole genome assemblies are still rare; moreover, many microeukaryotes possess their own microbiome, often resulting in high levels of contamination in transcriptomic samples. The resulting incongruences lead to conflicting and often spurious tree topologies that can be mitigated by careful selection of taxa and thorough curation of data (11, 12). Another issue in studies of diverse eukaryotes is the frequent reuse of gene families, and even orthologs in concatenated analyses (e.g., references 13–16), as this violates the assumption of independence that lies at the heart of phylogenetics (17–19). The EukPhylo pipeline addresses this non-independence by allowing users to select from a database of ~15,000 conserved gene families and then to automate ortholog selection for concatenation.

The recent increase in molecular data and bioinformatic methods has spurred the creation of numerous pipelines to infer homology, multisequence alignments (MSAs), gene trees, and species-level phylogenies (e.g., references 20–23). These phylogenomic tools differ in their intentions, allowed inputs (e.g., GenBank vs user-generated data), and outputs (e.g., MSAs, trees); yet few include the type of curation needed for analyses of data from microbial eukaryotes given issues with contamination (i.e., from microbiomes and environmental sequences). The first step of many pipelines is to collect homologous sequences, which can be gathered directly from public databases such as GenBank (24), Pfam (25), or OrthoDB (26). Many recent pipelines rely solely on BLAST (27) or other similarity-searching algorithms (e.g., USearch [28], VSearch [29], and Diamond [30]) to infer homology. However, BLAST is based on similarity only and does not take into account biological relationships (31), and further processing is necessary to confidently establish the source of sequences as well as homology.

Phylogenomic pipelines generally include an MSA step, which can be challenging when dealing with data from diverse eukaryotes that span ~1.8 billion years of evolution (32). For the subsequent estimation of species trees, recent phylogenomic approaches include methods that use gene trees as inputs in inferring species-level relationships (33). Such methods have been used for projects like the Open Tree of Life (34), and in studies of plants (35), animals (36), and viruses (37). An example of pipelines that follow these general steps is NovelTree, which performs homology assessment and gene tree construction, though it only accepts protein sequences as input data (20). Other examples that accept nucleotide sequences are PhyloTa (23), which focuses on homologous identification and collection, and Sumac (22), which focuses on building phylogenetic supermatrices. PhyloFisher is a pipeline that allows users to add new data to a manually curated set of 204 genes that have been used in estimating eukaryotic relationships, but it does not easily enable *de novo* (a.k.a. “on the fly”) exploration of contaminants, sequence statistics, or alternative gene families (38).

The importance of a taxon-rich data set for estimating phylogeny accurately is well established (39, 40), and adding diverse lineages (e.g., taxonomic position, rates of evolution, levels of missing data) can improve estimates of species relationships (41–44). However, even recent estimates of the EToL rely on relatively few taxa (e.g., 234 taxa in Burki et al. [45], 186 taxa in Al Jewari and Baldauf [46], 158 taxa in Cerón-Romero et al. [47], and 109 taxa in Strassert et al. [48]), and many groups now resample the same genes/data matrix in generating species trees (15, 45, 48, 49). The availability of user-friendly tools that facilitate the parallel processing of large numbers of taxa, therefore, has the power to increase the accuracy of large-scale estimates of eukaryotic phylogeny.

Here, we present EukPhylo version 1.0, a phylogenomics pipeline that supports taxon-rich analyses of gene families and gene trees through extensive data curation and that includes a suite of standalone tools plus curated databases. EukPhylo, parts of which are based on a pre-existing pipeline PhyloToL (50, 51), includes two main components, which we refer to as EukPhylo parts 1 and 2. EukPhylo part 1 takes input sequences from whole genome or transcriptome assemblies, applies several curation steps, and provides initial homology assessment against a customizable database of reference sequences to assign GFs. EukPhylo part 1 outputs curated coding sequences (CDSs) with gene families assigned, as well as a data set of descriptive statistics for each input sample. EukPhylo part 2 is highly modular: for a given selection of taxa and GFs, it enables homology assessment and produces MSAs by iterating the external tool Guidance (52, 53). From MSAs, EukPhylo part 2 builds gene trees and then includes an innovative workflow for tree topology-based contamination removal.

In addition to presenting the core pipeline, we describe the results of an example analysis of 500 conserved GFs from 1,000 taxa, demonstrating how EukPhylo allows users to explore how varying gene sets, taxon sets, or criteria for contamination removal lead to different biological inferences (e.g., differentiating host vs contaminant material, phylogeny). We also provide a suite of stand-alone tools that describe tree topologies and demonstrate the effectiveness of our novel tree-based contamination removal methods in improving tree topologies by assessing the monophyly of clades (e.g., ciliates, dinoflagellates, and metazoa) supported by robust synapomorphies as well as larger taxa (e.g., Amoebozoa, Archaeplastida, Opisthokonta, and Stramenopiles, Alveolata, Rhizaria [SAR]).

## RESULTS

We divide our results into three sections: (i) a broad overview of EukPhylo v.1.0; (ii) a section on performance of the core pipeline that focuses on the power of the approach through a description of part 1 (gene family assignment), part 2 (MSAs and single-gene trees), and utilities; and (iii) a section on the performance of the contamination loop accompanied by a case study of phylogenomic analyses of 1,000 diverse species and 500 genes for which we build species trees at each of four stages of the contamination loop. For the latter, our intent is to emphasize the power and transparency (i.e., in recording “rules” and retaining removed sequences) of EukPhylo for analyzing complex data generated for uncultivable microbial eukaryotes.

### Overview: the pipeline and accompanying scripts

EukPhylo v.1.0 is a flexible and modular pipeline that enables efficient phylogenomic analysis of eukaryotes and includes phylogeny-informed curation of ’omics data. Compared to its predecessor PhyloToL (50, 51), EukPhylo v.1 streamlines the workflow for assigning gene families to data from transcriptomes and genomes (EukPhylo part 1), expands options for data curation both before and after producing MSAs and gene trees (EukPhylo part 2), and provides an extensive set of utilities that can be used within or independent of EukPhylo. To supplement the power of EukPhylo, we publish several accompanying databases, described below. All components of the toolkit are written in Python and are available for download on GitHub (https://github.com/Katzlab/EukPhylo) and Zenodo (DOI:10.5281/zenodo.13323372), and we provide a containerized version through Docker; the GitHub site also includes a detailed user manual and quickstart guide. All references below to files available through Figshare refer to this Figshare page: https://figshare.com/projects/EukPhylo_Supplemental_Files/196552.

#### The core pipeline (parts 1 and 2)

**Fig 1:**
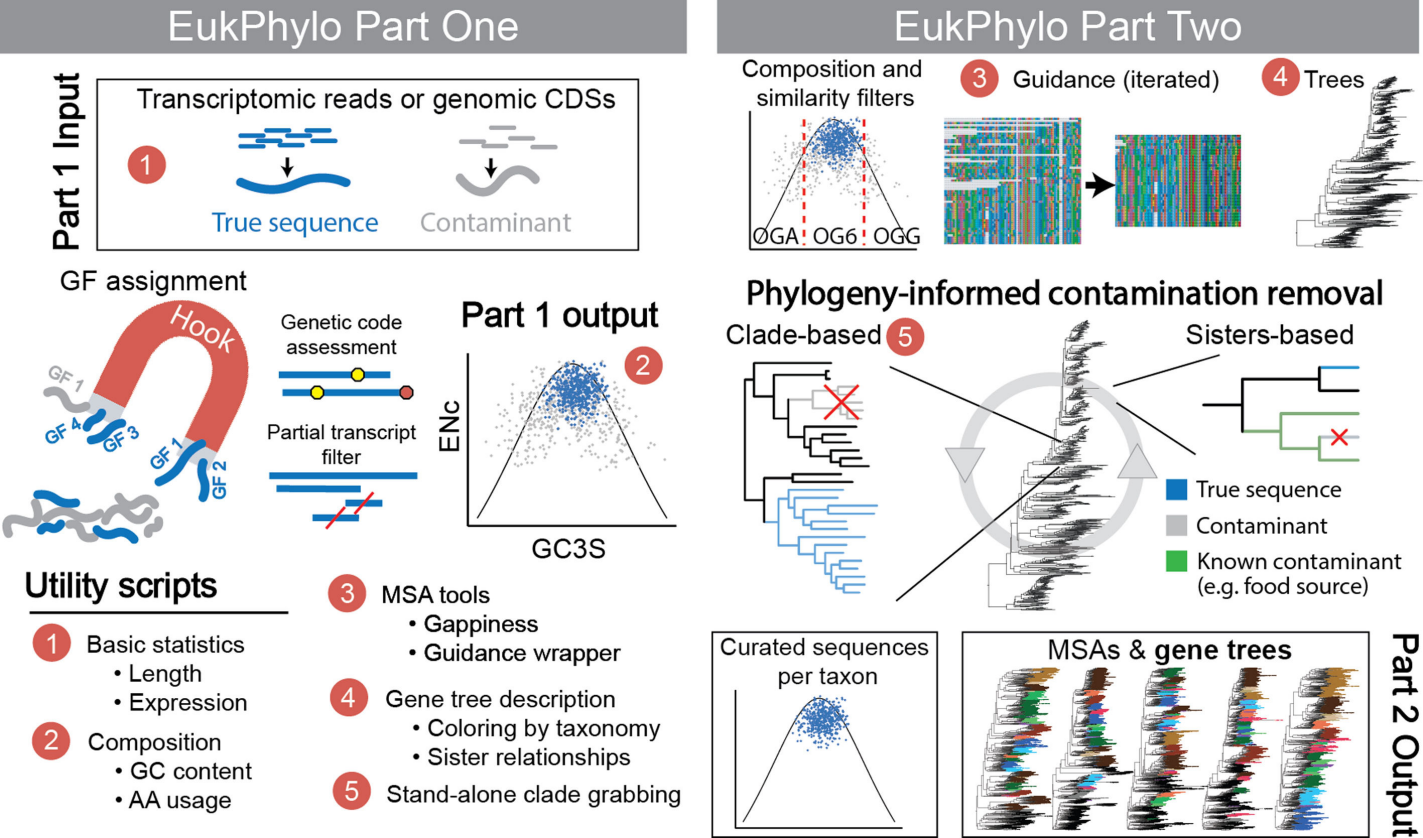
A schematic of the EukPhylo v.1.0 core pipeline. EukPhylo comprises two main components, part one and part two. Part one is primarily intended to apply preliminary filtration steps and assign gene families using a reference database. This reference database can be the Hook version 1.0 as described in the main text, or a custom database. Part one takes as input assembled transcripts or genomic CDS and is able to flexibly handle a variety of genetic codes. In the graph under the “Part 1 output” heading, we show using silent site GC content (GC3S) vs the effective number of codons (ENc) that the true sequences from the sequenced sample (blue) tend to have similar composition, with contaminant sequences (gray) falling outside of this range (red dashed lines under Part Two) represent user-selected cutoffs for removing putative contaminant sequences based on GC3S; see Supporting Information). Part two generates MSAs by iterating Guidance (52, 53) multiple (by default, five) times for rigorous homology assessment of each gene family, and then builds gene trees. We present a novel phylogeny-informed approach to contamination removal, where contamination is removed from trees in an iterative fashion, either by keeping only sequences in robust clades (“Clade-based”) or removing sequences sister next to known contaminants (“Sisters-based”). We also exemplify the suite of utility tools accompanying this core pipeline, identified by numbers (red circles) where the tool can be applied.

EukPhylo is designed to take as input assembled transcripts, genomic CDSs, or any sequences with names matching simple criteria (i.e., a 10-digit taxon code plus a unique identifier) as described in Materials and Methods (see also Supplemental Material). Curation steps are built into both parts of the pipeline, first enabling analysis of data within a taxon based on sequence properties (e.g., GC content, codon usage; see Table S1 and File S3 at https://figshare.com/projects/EukPhylo_Supplemental_Files/196552) and then using homology assessment by Guidance (52, 53) and phylogeny-informed removal of contaminants (Fig. 1). EukPhylo part 1 allows users to use either the built-in Hook Database of ~15,000 eukaryotic GFs or a user-supplied custom database to produce curated amino acid and nucleotide sequences for each taxon (Fig. 1). EukPhylo part 2 takes these files as input and constructs an MSA and gene tree for each gene family (Fig. 1). EukPhylo part 2 also includes a novel workflow for phylogeny-informed contamination removal that we refer to as the “contamination loop,” which identifies both likely contaminant sequences and most robust clades (i.e., “clade grabbing”) and writes putative contaminant sequences out into a file that users can publish to increase the transparency of their curation methods.

#### The contamination loop

We provide an additional overview of the “contamination loop” included in EukPhylo part 2 as it is among the more unique features and is particularly important for the curation of transcriptome data from uncultivable microeukaryotes. This contamination loop has two modes, both of which rely on user-defined rules. The first is “sister/subsister” removal, in which single sequences are removed based on their taxonomic position in single-gene trees, and which can be implemented with a requirement that these putative contaminants sit on short branches. The second is “clade grabbing,” which retains sequences for which we have greatest confidence based on taxonomic density in single-gene trees. Examples where sister and subsister rules are applicable include cases where a taxon, or a pair of taxa, is contaminated by a food source or by a known host as in the case of parasites. The clade grabbing mode is applicable for well-sampled taxonomic groups that form sizable clades in single-gene trees, and in this mode sequences that do not fall into clades of a user-defined size are removed. We note that this process likely removes a considerable amount of vertically inherited data by retaining only the most robust clades and hence should be used with caution in studies that focus on the history of individual genes. EukPhylo includes a set of scripts (e.g., ContaminationBySisters.py and CountTaxonOccurrence.py; see Materials and Methods) that help users to assess taxon presence and sister relationships across single-gene trees to establish sets of rules to use in each mode of the contamination loop. We believe that EukPhylo’s ability to document both rules and sequence choice in a transparent manner is a substantial improvement to best practices in the field, and we exemplify the effect of the contamination loop in estimating EToL in the final section of the results.

#### Databases

**Fig 2:**
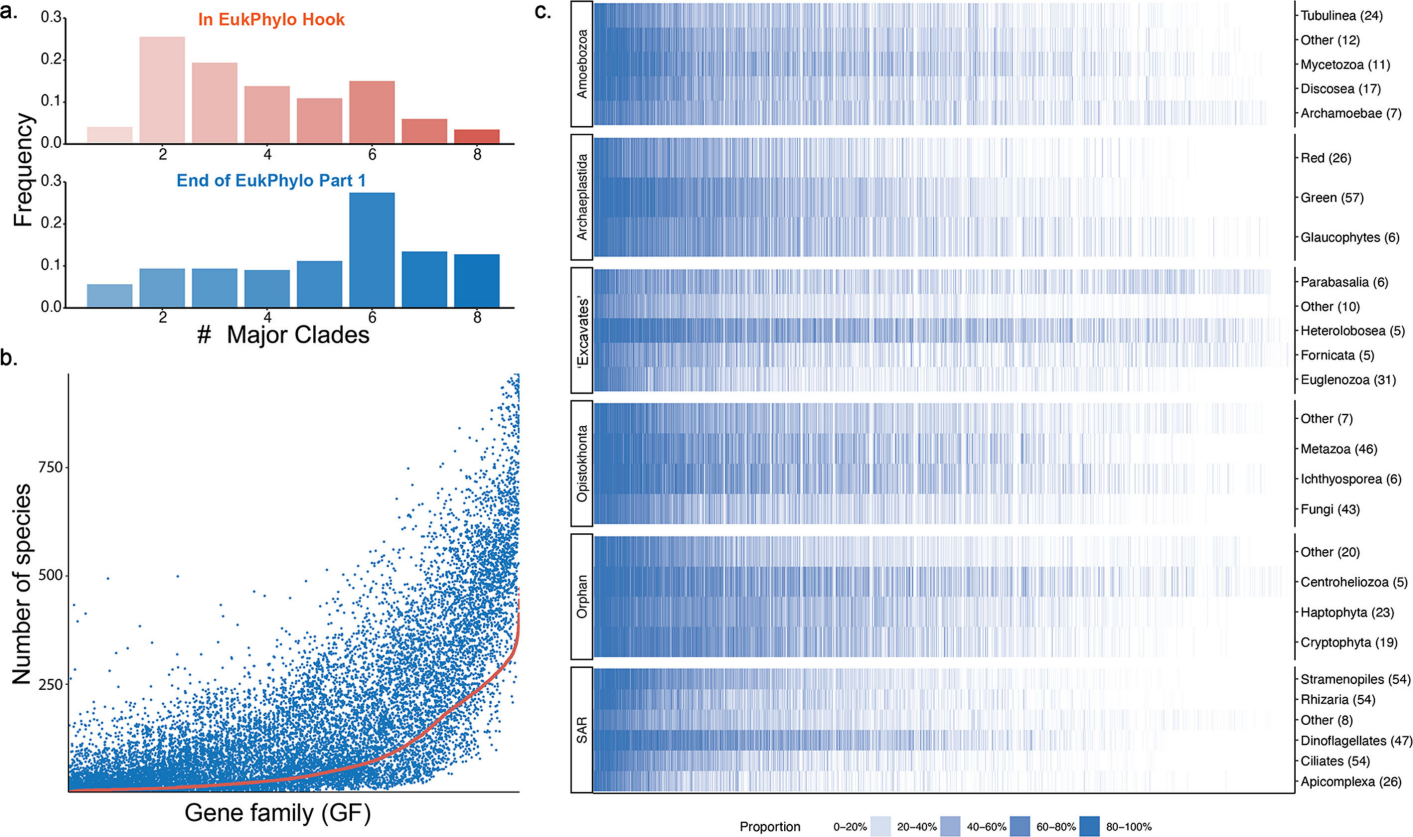
The Hook reference database, which is used in EukPhylo part 1, effectively captures taxonomic diversity in GF assignment. We divided the taxonomic diversity among eight major clades, of which six are eukaryotic. While most GFs as represented in the Hook database are only present in two to four major clades, once assigned to our more diverse data set, most GFs are present in five to eight major clades, with a mode of 6 (the number of eukaryotic major clades) (a). The number of species with a GF in the ReadyToGo files (blue) correlates with the number of species with that GF in the Hook (red), with very few GFs losing diversity (b). Panel **c** describes the proportion of species (intensity of color) in each of the six eukaryotic major clades (each row represents a minor clade, and the rows are grouped by major clade) in which each GF is found. Each column represents a GF; those found in more species are on the left, and those with fewer species are on the right.

To provide an option for users interested in exploring data from a limited number of species (e.g., transcriptomes or genomes generated by their research groups), we provide several taxon-rich databases aimed at analyses of eukaryotic phylogeny: (i) our Hook reference database of ~15,000 proteins for GF assignment; (ii) files for 1,000 species containing amino acid and nucleotide sequence that have been assigned to these GFs (called ReadyToGo or R2G files), which we use in our assessment of the performance of EukPhylo described below; and (iii) curated MSAs and trees for 500 conserved gene families. The Hook Database is composed of 1,426,763 sequences across 15,138 GFs (see Table S3 and File S1 at https://figshare.com/projects/EukPhylo_Supplemental_Files/196552). It captures a broad diversity of eukaryotic gene families and was built starting from OrthoMCL version 6.13 (54), which we sampled to select for GFs that are present across the eukaryotic tree and/or present in undersampled lineages of eukaryotes (see Materials and Methods; Fig. 2, Fig. S1). To add value for users, we also include functional annotations for each GF in the Hook (Table S4 at Figshare; see methods in Supporting Information). Alternatively, users can insert their own Diamond-formatted database in lieu of the Hook, to target only specific genes of interest; in this case, we encourage users to include some housekeeping genes (e.g., actin, HSP70) as controls.

To develop an exemplary taxon set for users, we chose 1,000 species, balancing taxonomic diversity and data quality and focusing on diversity of eukaryotic lineages. Starting with more than 2,500 genomes and transcriptomes (from public databases and our own sequencing effort), we used the EukPhylo toolkit to choose 1,000 species based on data quality (analyzing the GC content at the silent site fingerprint and phylogeny-based identification of proportion of contamination as a proxy for quality) and taxonomic representation. The 1,000 species include 628 eukaryotes, of which 199 are represented by annotated genome sequences and 429 by transcriptome data (44 coming from our own sequencing effort; Table S1 and S7 at Figshare). This set of eukaryotes emerged from pilot analyses that aimed to maximize taxonomic representation with the best available data at the time of the launch of the project. We also include 275 bacteria and 97 archaea, all of which have whole genome sequences (Table S1 and S7 at Figshare). As described in more detail in the Materials and Methods section, each species is represented by a 10-digit code that captures taxonomy, at least as understood when the data were first processed. For example, humans are coded as Op_me_Hsap (Op for Opisthokonta, me for metazoa, and Hsap for *Homo sapiens*) and *Arabidopsis thaliana* as Pl_gr_Atha (Pl for Archaeplastida, gr for green algae).

#### Utilities

Besides the main pipeline, EukPhylo includes a set of stand-alone utility scripts that aim to increase the power of analyses done with or without the core EukPhylo pipeline. We divide these scripts into five main categories: basic statistics, composition tools, MSA tools, gene tree description, and contamination removal (Table S2 at Figshare), and we provide details of each on the GitHub wiki. The EukPhylo utilities can be used with outputs from the pipeline, or with external files (generally fasta files and/or Newick strings), so long as taxon names have been modified to match the 10-digit criteria used by EukPhylo. Examples of such utilities include a script to calculate the “sharedness” of gene families across taxa, which allows users to identify focal gene families for each study, as well as tools for coloring and relabeling gene trees, which can be very helpful in exploring taxon-rich data and generating figures for publication.

### Performance of the core pipeline

We provide our description of performance in two sections that reflect the two major parts of EukPhylo (part 1: GF assignment; part 2: generation of MSAs and trees), and for each, we start with a brief description of the computation resources needed before moving into specifics of the tool. We demonstrate the performance of part 1 with an analysis of data from 1,000 species. We then demonstrate part 2 on a select set of 500 gene families from the output of part 1, focusing on generation of MSA and initial gene trees. We discuss the contamination loop in the “performance of the contamination loop” section below.

#### EukPhylo part 1

##### Computational resources

To benchmark the resources needed for EukPhylo part 1, we compared the speed in processing assembled transcriptomes and genomes through to “ReadyToGo” files for EukPhylo part 1. Using a desktop computer (iMac Pro 2017, 64GB of RAM, 10 cores) and a high-performance computing cluster (HPC; 128 GB of RAM, 24 cores), we processed 10 and 100 transcriptomes and genomes (Table S5 at https://figshare.com/projects/EukPhylo_Supplemental_Files/196552). As expected, processing genomes with EukPhylo part 1 was considerably faster compared to the transcriptomes on both computers as coding domains are already called for genomes. On the desktop computer, it took roughly 2 h for 10 transcriptomes (513,904 transcripts) and 24 h for 100 transcriptomes (3,294,484 transcripts), while the same data sets took 2 and 16 h, respectively, on the HPC. Processing the genomes, it took 1 h 20 min and 24 h on the desktop computer for 10 (106,249 CDS) and 100 taxa (1,158,224 CDS), respectively, and 25 min and 21 h on the HPC to run the same set of taxa. This demonstrates the feasibility of running EukPhylo part 1 on desktop or even laptop computers if an HPC is not available.

##### Gene family assignments with optional curation for composition

To demonstrate the capabilities of EukPhylo for exploring eukaryotic GFs, we assigned sequences from our 1,000 focal taxa (Table S7 at Figshare) to GFs from our Hook Database using EukPhylo part 1. Despite the fact that the starting OrthoMCL database is biased in terms of taxonomic availability (e.g., biased toward parasitic lineages [54]), the 15,138 GF Hook Database assigned gene families to a broad diversity of taxa, including poorly represented taxa like *Telonema,* Centrohelidae, and other orphan lineages (labeled EE for “everything else”; Fig. 2; Table S1 at Figshare). In fact, the taxonomic distribution of major clades in our ReadyToGo files is greater than in the Hook itself, with more than 75% of the GFs present in at least four major clades in the ReadyToGo files (Fig. 2a), and with an increase in the number of species per GF in the ReadyToGo files (blue dots on Fig. 2b) compared to the Hook (red line on Fig. 2b), demonstrating the power of EukPhylo to assign gene families to a great diversity of taxa. Nevertheless, the distribution of GFs across taxa is highly variable, reflecting at least three phenomena: the differences between transcriptome and whole genome data, the prevalence of gene loss in some lineages (e.g., fungi [55] and parasites), and the challenges of identifying fast-evolving homologs using default Guidance and BLAST parameters (Fig. 2c). Users can address the latter difficulty, which could give rise to “false negatives” (i.e., divergent sequences being excluded from the analysis because they were not assigned a GF or were assigned the wrong GF), by (i) adjusting parameters such as the BLAST e-value for GF assignment in EukPhylo part 1 or the Guidance sequence removal cutoff in part 2; and/or (ii) customizing the reference database for GF assignment to contain examples of fast-evolving homologs.

Using EukPhylo utilities (CUB.py, GC_identifier.py), we further refined data based on taxon-specific GC content ranges (see Supporting Information) to produce ReadyToGo files with sequences labeled by composition (OG6 if in GC3S range for each species, OGG and OGA if more GC-rich or AT-rich, respectively). This is possible as each organism tends to use G+C (as opposed to A+T) at a particular average proportion; GC content among genes within eukaryotic genomes tends to vary in a relatively narrow range, particularly at silent sites (56, 57). Therefore, a wide range of GC content within a sample of coding sequences is likely to denote that signal from multiple organisms (i.e., contamination) is being captured. This is the same theory behind widely used contamination assessment tools such as BlobToolKit (58), though we explore both composition and codon usage through our toolkit (e.g., CUB.py, 57).

We provide the resulting ReadyToGo databases containing sequences from the focal 1,000 species that match the ~15,000 gene families in the Hook Database. These data can be used by researchers interested in efficiently placing species into a broad phylogenomic context. Among eukaryotes, the average number of sequences per species that are assigned to Hook gene families is 6,681, and these fall among 3,287 gene families. The numbers are smaller for bacteria and archaea, with an average of 1,804 and 1,233 sequences being assigned to 1,274 and 948 gene families, respectively (Table S1 and S3 and File S2 at https://figshare.com/projects/EukPhylo_Supplemental_Files/196552). The use of a relatively relaxed e-value cutoff of 10^−5^ ensures that we capture putative homologs from eukaryotes that have elevated rates of evolution (e.g., parasites), and we improve homology inferences with Guidance (52, 53), the tool we use for MSA reconstruction as described in part 2 below.

### EukPhylo part 2

#### Computational resources

To benchmark the resources needed to run EukPhylo part 2, we measured the time required for processing gene families for both the pre- and post-contamination removal stages, as well as with and without a “blacklist” (i.e., non-homologs removed by previous runs Guidance). Using a high-performance computing cluster, we processed 50 of the 500 GFs in our pilot analysis, carefully tracking run times (Table S5 at https://figshare.com/projects/EukPhylo_Supplemental_Files/196552). On the HPCs, it took roughly 25 h to get to the first trees for the 50 GFs using an array (1 GF per job), and then 44.5 h to run the phylogeny-informed contamination removal process (50 GFs per job, Table S5). Using a blacklist improved running time considerably: 3.25 h to produce aligned files compared to 9 h without (Table S5). Concatenation within EukPhylo after the post-contamination removal stages for the 50 OGs was fast, taking only 15 min. Note that for these analyses, we used the same versions of programs as our pilot study described below with the exception of Guidance, for which we used an updated version (v.2.1 as available on GitHub, accessed 17 June 2024). We ran comparisons and found that this newer Guidance version (2.1) produced similar results as the older version (v.2.0.2), but more efficiently (likely because the new version is parallelizable). Given this, we have updated EukPhylo to include this newer Guidance on Github and Zenodo.

#### Initial MSAs and single-gene trees

To demonstrate the power of EukPhylo in estimating gene family membership, we selected 500 gene families based on taxonomic presence using two criteria: (i) they are among the most shared GFs in our 1,000 taxa and (ii) these GFs have relatively low paralogy, making analyses more efficient; both parameters estimated using the SharedOGs.py utility script. Starting with the 1,000 ReadyToGo files generated by EukPhylo part 1 (see above), we ran EukPhylo part 2 to generate 500 MSAs and single-gene trees (Table S13 at https://figshare.com/projects/EukPhylo_Supplemental_Files/196552). We choose to use the “similarity filter” with an amino acid identity cutoff of 99% to remove highly similar sequences (e.g., recent paralogs, alleles) within species as a means of shortening processing times (see Supplemental Methods). EukPhylo generates MSAs using Guidance (52, 53), which we also use as a filter to remove putative non-homologs. In our analyses of 500 GFs across 1,000 species, 15,486 sequences out of 581,539 (2.66%) were removed by Guidance as putative non-homologs (File S7 at Figshare). We offer several options for tools to build single-gene trees from the resulting MSAs, which are easily configured by the user when running the pipeline. The default option is the “fast” mode of IQ-Tree (59), and we include other modes of IQ-Tree, as well as RAxML (see methods and manual on GitHub), or users can stop EukPhylo after MSA generation to use other phylogeny programs.

### Performance of the contamination loop assessed by species tree estimates

**Fig 3:**
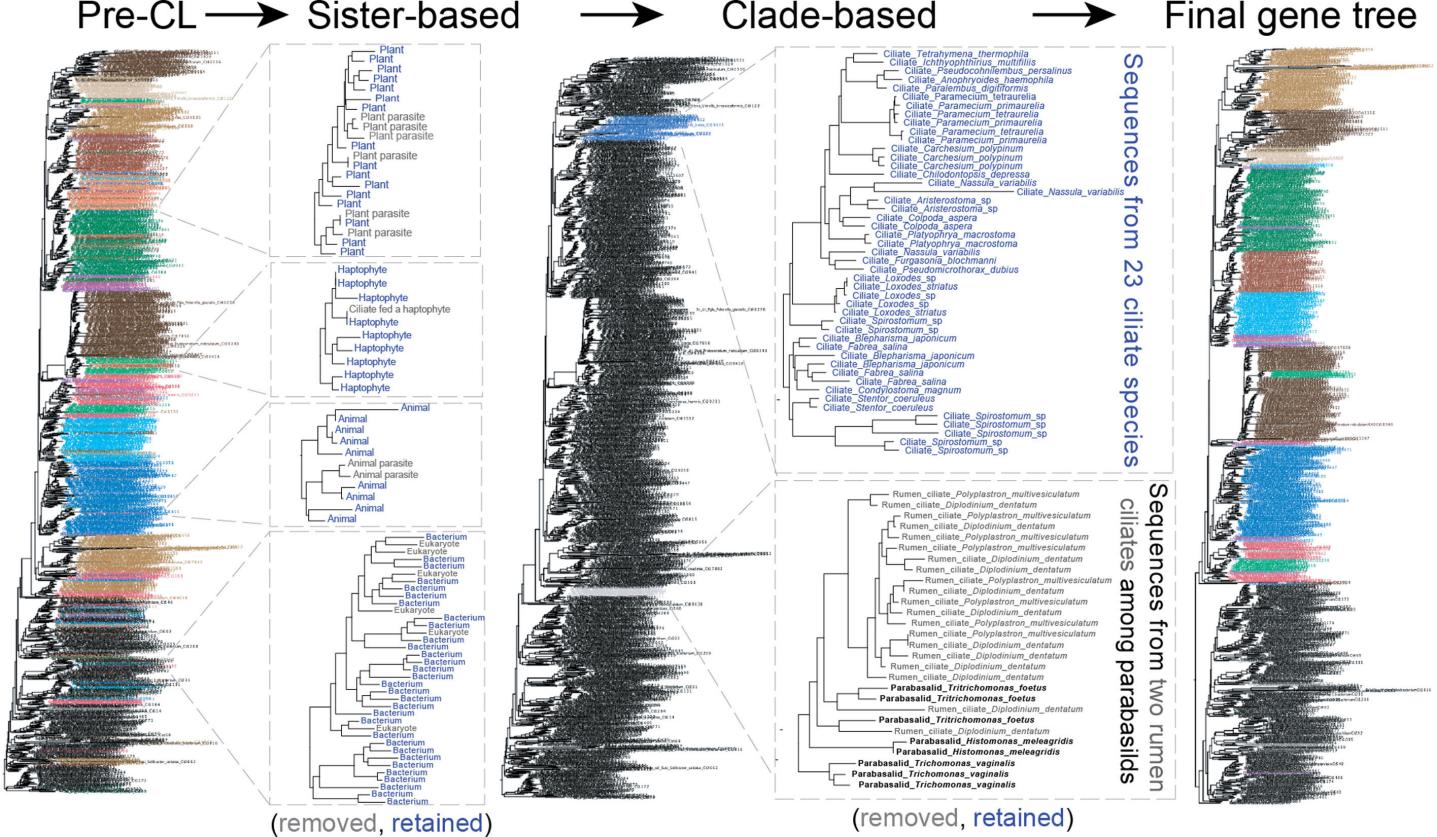
A cartoon depicting phylogeny-informed contamination removal, which is a component of EukPhylo part 2. Users can use the contamination loop to iteratively remove sequences based on their sister species in single-gene trees. Depicted on the pre-contamination loop tree are sequences (gray) that are either from hosts in analyses of parasites (upper left) or bacterial sequences that come as contaminants in analyses of eukaryotic transcriptomes (lower left). In a second method of contamination removal, users can “grab” (retain) sequences falling in monophyletic clades that meet user-specified robustness criteria (e.g., minimum target group species count and maximum number of non-group species). In the case depicted here, we identified substantial contamination of a subset of ciliate transcriptomes by parabasalids with which the ciliate species are known to share an environment (cow rumen). To remove this ciliate contamination, we used EukPhylo to retain only ciliate sequences falling in clades with at least 12 species; an example of a retained clade is given in blue, and a removed clade in gray in the bottom right.

**Fig 4:**
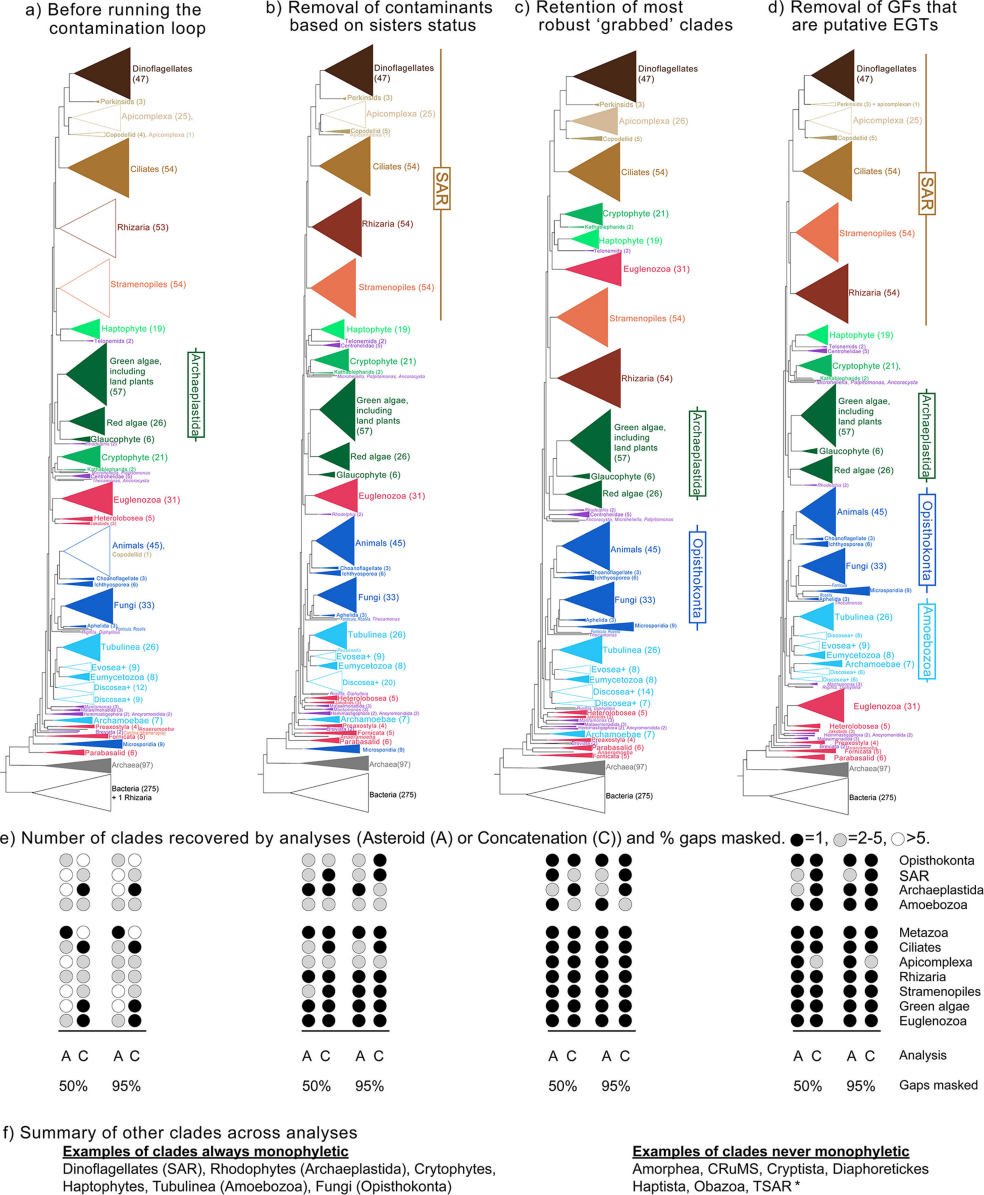
(**a–d**) Concatenated analyses (50% gap trimming) at four stages in the contamination removal process are generally concordant with published hypotheses as most morphologically defined clades (e.g., dinoflagellates, green algae, Euglenozoa, stramenopiles) are recovered consistently. Filled triangles indicate that the labeled group is monophyletic; empty triangles indicate non-monophyly. The four analyses are (a) before contamination loop, (b) after removal of contaminants based on sister/subsister rules; (c) after clade grabbing to keep “best” sequences; and (d) after removal of trees possibly affected by both primary and secondary EGT. Notably, the monophyly of Opisthokonta emerges after clade grabbing (c) while the monophyly of Amoebozoa and SAR only appears after removing trees affected by EGT (d). Some “orphan” lineages (purple) are stable across trees (i.e., telonemids are always sister to haptophytes, breviates are always toward the root) while other lineages (e.g., Centrohelidae, Hemimastigophora + Ancyromonida) move position across trees; this likely reflects a combination of the effects of missing data and a lack of close relatives. We only mark Archaeplastida as monophyletic when the “orphan” taxon *Rhodelphis* falls sister to or within this clade. Across each stage of the contamination removal process, the number of key eukaryotic groups that are monophyletic increases in both Asteroid (a) and concatenated (c) analysis, removing sites that are either 50% and 95% gaps (e). Finally, we report groups that are always monophyletic and others that are never found (**f**); * indicates that TSAR is recovered only in the Asteroid analysis (50% gap trimmed) after clade grabbing.

In this section, we first describe the specifics of contamination removal for the analyses of 500 gene families sampled from 1,000 species. Then we estimate EToL at four stages to demonstrate the performance of the phylogeny-based contamination removal tool built into EukPhylo (Fig. 3). The four stages are (i) before the contamination loop (Fig. 4a); (ii) after applying sister-based rules to iteratively remove sequences determined to be potential contaminants based on user-established rules (Fig. 4b); (iii) after clade grabbing by retaining only sequences for which we have the greatest confidence based on user-established expectations of taxon density (Fig. 4c); and (iv) after removing gene families that include putative endosymbiotic gene transfers (EGTs, Fig. 4d). All files related to this analysis, for each step of the contamination loop, can be found on Figshare as a demonstration of another aspect of EukPhylo: the ability to easily retain intermediate files and track removed sequences.

#### Applying the contamination loop

##### Sisters/subsisters removal

EukPhylo allows users to remove sequences that may be contaminants (e.g., in single-cell transcriptomes contaminated by food sources) by setting rules, which can include a requirement for short branches. To demonstrate this phylogeny-informed contamination removal, we set sisters/subsister rules based on our knowledge of the biology of the taxa plus inspection of single-gene trees generated for 500 GFs and 1,000 species. For example, we set a rule to remove sequences from the ciliate *Favella* (Sr_ci_Fehr) when it falls sister to a haptophyte (EE_ha), a known food source (Table S8at https://figshare.com/projects/EukPhylo_Supplemental_Files/196552; Fig. 3). We also set “blanket” sister rules, removing any single species from well-sampled clades (e.g., ciliates, animals) that fall sister to bacteria or archaea regardless of branch length; for less-well-sampled clades (e.g., haptophytes and cryptophytes), we removed single sequences only if they fell on short branches (i.e., 0.5 times the average node-to-tip distance for a given tree) sister to bacteria or archaea (Table S8 at Figshare). Such an approach is an efficient way to remove the contamination in ’omics data sets, but also should be used with caution given the limited power single-gene trees have in estimating eukaryotic phylogeny. In addition, this approach has the potential to remove recent lateral gene transfers and hence should be used with caution in studies asking questions about individual genes.

EukPhylo also allows the removal of sequences that are “co-contaminants,” affecting pairs of sequences. As an example of this, inspection of individual gene trees showed that the transcriptomes of fungus-like species *Aphelidium insulamus* and *Aphelidium tribonematis* (Op_ap_Ains and Op_ap_Atri) are highly contaminated and frequently branch together among lineages including stramenopiles and Amoebozoa; we therefore infer that these taxa are contaminated by the same sources (perhaps in laboratory preparation or in sequencing), so we use the “subsisters” option to remove these sequences when together they fall sister to non-Opisthokonta. After applying both sister and subsister rules (Table S8 and S9 at Figshare, respectively), the greatest proportion of removed sequences is from taxa within the major clade SAR (abbreviated as Sr), which includes a majority of the field-caught single-cell transcriptomic samples (ciliates, foraminifera) from our lab (File S8 at Figshare). At the end of the sisters mode of the contamination loop, EukPhylo removed 50,903 of 565,225 sequences (Table S13 and File S8 at FigShare). Importantly, all removed sequences are recorded for anyone interested in tracking specific cases.

##### Retaining “best” sequences by clade grabbing

The second type of phylogeny-informed contamination removal allows users to retain sequences with the greatest confidence based on their presence in robust clades (Fig. 3), again using user-defined rules that can be easily shared on publication of analyses. We first ran ‘clade grabbing’ only for ciliates (a clade whose monophyly is not controversial) as we had a strong signal of contamination of parabasalid sequences putatively mislabeled as ciliate from species isolated from the digestive system of cows; here, ciliate transcriptomes containing parabasalid sequences (Ex_pa ) cause the ciliates to spuriously fall near parabasalids (Fig. 3, center). After addressing the high level of contamination of ciliate data, we deployed “clade grabbing” more broadly using clade sizes determined empirically based on the EukPhylo utility script CladeSizes.py (see Materials and Methods). For example, given that we have a total of 45 diverse metazoans, we kept only clades containing at least 11 metazoan species (i.e., Op_me); here, we allow up to 10% of a clade to be non-metazoan species to account for long-branch orphan lineages that “wander” in single-gene trees (Table S1 and S6 at Figshare).

Clade grabbing works best for well-sampled clades and should be used with caution, for example when considering sequences from orphan lineages. Given this, we identified a list of “exceptions” (i.e., taxa with few close relatives in our analyses; for example, orphan lineages such as *Mantamonas* and *Hemimastix*) for which all sequences are retained independent of clade size; these lineages lack robust sisters in our analyses, so the topology of single-gene trees does not inform the robustness of sequences from these taxa. In the end, clade grabbing removed 129,458 of 514,272 sequences, and we share rules and sequences in the Supplemental Material material. Here, again, we emphasize the importance of transparency and user-defined rules for clade grabbing, as this process will most likely remove “good” sequences as well as contaminants, while selecting for the strongest signals in single-tree topologies.

##### Removing gene families that may be affected by EGT

Given the many papers demonstrating a substantial effect of EGT from plastid genomes to nuclear genomes (reviewed in reference 60), we conducted a final analysis by removing gene families that may have been affected by primary and/or secondary EGT. This provides an example of how the methods built into the contamination loop can be extended beyond their basic applications. To start, we used the utility scripts CladeGrabbing.py and CladeSize.py to identify gene families with a putative photosynthetic history; here, we define gene families possibly affected by primary EGT as gene families with photosynthetic lineages nested among bacteria (often cyanobacteria, but allowing other bacterial sisters given the prevalence of gene loss and lateral gene transfer [LGT] among bacteria) while secondary EGTs are identified as gene families with the greatest proportion of sequences of intermingled photosynthetic lineages (e.g., dinoflagellates nested among diatoms; Table S12 and File S5 at Figshare). After removing 169 GFs possibly affected by primary and/or secondary EGT, respectively, we ran concatenated and Asteroid (61) analyses, and we compared the resulting “EGT removal” trees below .

### Inferring EToL at four stages

To demonstrate the power of the contamination loop, we discuss the topology of EToL inferred from before the contamination loop (Fig. 4a) plus three stages after deploying contamination loop tools (Fig. 4b through d). For these analyses, we generated both a concatenated alignment using EukPhylo’s concatenation feature, which aims to select the most robust orthologs based on density of close relatives (see Materials and Methods), and an Asteroid tree (61); for all alignments, we masked columns to remove those with ≥95% and ≥50% missing data. As a measure of robustness, we focus on the presence of clades whose monophyly is supported through ultrastructure and/or by robust synapomorphies (e.g., ciliates, dinoflagellates, metazoa, fungi, green algae), as well as higher-level taxa (e.g., Amoebozoa, Archaeplastida, Opisthokonta, SAR). Our phylogenomic results are subject to numerous caveats, described below, and the importance of this section lies in the comparison across stages of contamination removal rather than inferences about the structure of EToL. We find that tree topologies are generally concordant through the various stages of our analysis of 1,000 species, though with marked improvements through data curation (Fig. 4a through e, with monophyletic clades represented by filled triangles [a–d] or filled circles [e]; Fig. S3; Table S12 at https://figshare.com/projects/EukPhylo_Supplemental_Files/196552).

The estimate of EToL prior to the contamination loop is largely consistent with the published literature (Fig. 4a and e) as our taxon-rich approach recovers many clades with robust synapomorphies across all analyses (50% and 95% gap trimmed, concatenated, and Asteroid), including fungi, dinoflagellates, cryptophytes, haptophytes, and Tubulinea (Fig. 4a and e; Table S12 at Figshare). However, the monophyly of some clades is disrupted by single species: the non-monophyly of Rhizaria is due to the placement of the foraminifera *Notodendrodes hyalinosphaira* (Sr_rh_ArpA) among bacteria, and the parasite *Piridium sociabile* (Sr_co_Psoc) falls within animals in concatenated analyses prior to contamination removal (Fig. 4a; Table S12 at Figshare). Other aberrant observations include the placement of Microsporidia (a lineage known to have elevated rates of evolution [62, 63]) and Archamoebae (another parasitic lineage) toward the base of the eukaryotic portion of the tree. Asteroid (61) analyses of these data reveal further evidence of contamination as numerous clades (e.g., ciliates, Euglenozoa, green algae; Table S12 at Figshare) are non-monophyletic, which highlights the impact of contamination in ‘omics data from microeukaryotes.

The iterative contamination removal in EukPhylo improves tree topology as we remove contamination based on user-set sister/subsister rules and then retain only the most robust sequences through clade grabbing (Table S8 and S9 at Figshare). Deploying rules for sister-based contamination removal improves the topology of EToL in that we consistently recover clades like Metazoa, Euglenozoa, colpodellids, and Rhizaria (Fig. 4b and e; Table S12 at Figshare). However, the monophyly of ciliates, another clade with robust synapomorphies (cilia and dimorphic nuclei [1]), emerges only after deploying the second tree-based contamination method by clade grabbing based on only retaining clades with a pre-set number of target taxa (Fig. 4c; Table S10 and File S5 at Figshare). Here, clade grabbing allowed us to distinguish ciliate signal from contamination by parabasalids among a subset of ciliates isolated from the rumen of cows (see above). Our final curation step, “EGT removal,” excluded gene families that may be affected by primary and/or secondary EGT (Fig. 4d, Table S12 and File S5 and S6 at Figshare). Intriguingly, two members of the genus *Rhodelphis* fall sister to red algae only in concatenated analyses after EGT removal (Fig. 4d), consistent with previous analysis of these “orphan” species that are perhaps best considered part of Archaeplastida (64).

We also assessed changes in higher-level eukaryotic taxa throughout stages of contamination removal. Opisthokonta (animals, fungi, and their microbial relatives) emerges consistently only after sister/subsister removal for both Asteroid (61) and concatenated analyses (Fig. 4; Table S12 at Figshare). The monophyly of SAR (Stramenopila, Alveolata, and Rhizaria) and Amoebozoa is recovered in a subset of analyses following contamination removal (Fig. 4). The orphan lineage Hemimastigophora is consistently sister to the Ancyromonidida, falling nested among “excavate” and orphan lineages toward the root of our trees. The placement of other orphan lineages (purple branches, Fig. 4; Fig. S3; Table S9 at Figshare) varies across analyses, with some lineages like Breviata, Malawimonadida, and *Mantomonas* falling toward the root of EToL (Fig. 4; Fig. S3; File S9 at Figshare), though missing data likely confound the placements of all of these lineages (see below). Other proposed eukaryotic “supergroups” (e.g., CRuMs, Obazoa, Diaphoretickes) are not recovered in any analysis, and the proposed clade “TSAR” (*Telonema* + SAR) is recovered only in the clade-grabbed trees analyzed by Asteroid with 50% gap trimming (Fig. S3; Table S12 and File S9 at https://figshare.com/projects/EukPhylo_Supplemental_Files/196552).

## DISCUSSION

EukPhylo v.1.0 provides a platform for the efficient curation and analysis of ’omics data from eukaryotes, using phylogeny-informed methods that enable exploration of both gene families and species relationships. Key aspects of EukPhylo are its repeatability, flexibility, and transparency, as users can record parameters (e.g., in identifying contaminants) and report both retained and removed sequences through every step. Analyses of diverse microbial eukaryotes, and particularly uncultivable lineages characterized by single-cell ’omics, require curation to select the gene families most shared among focal species, identify homologs, and remove misidentified sequences (e.g., from contaminants and/or symbionts). In recent literature, numerous “boutique” approaches that require time-consuming hand curation have been used to estimate eukaryotic phylogeny from a relatively small number of gene families (e.g., references 13, 48, 65–67). Given the effort required here, some studies rely on resampling data (i.e., choosing orthologs to match previous concatenated gene sets), which can lead to issues arising from a lack of independence (reviewed in reference 68). While standards of curation and data quality have been developed for analyses such as genome assembly and annotation (e.g., references 69, 70), analogous standards do not yet exist for phylogenomics, and we believe that EukPhylo will in part fill this gap by providing transparent and repeatable methods.

EukPhylo provides a streamlined method for processing both genomic and transcriptomic data that enables users to maximize analytical power by choosing most shared gene families for each study, and to expedite data curation via both per-taxon and tree-based comparative approaches (Fig. 3). The databases and scripts require minimal effort for installation and are structured for users with modest bioinformatic skills, as accessibility and usability are key considerations in designing scientific tools (71). We provide EukPhylo with default settings for parameters that we believe are a reasonable starting place for analyses, though all parameters are easily customizable. Alongside the code, there is a comprehensive manual on GitHub that describes how to use the EukPhylo toolkit (https://github.com/Katzlab/EukPhylo-6/wiki). For those interested in a taxon-rich data set for analyzing data from previously uncharacterized taxa, we provide the EukPhylo Database, a set of curated data sampled from 1,000 diverse transcriptomes and genomes from eukaryotes, bacteria, and archaea (Table S1 and File S2 at https://figshare.com/projects/EukPhylo_Supplemental_Files/196552). Hence, EukPhylo enables large-scale phylogenomic analyses of eukaryotes.

EukPhylo’s modular nature allows users to stop and restart the pipeline at multiple points, add preferred methods that are not built into EukPhylo (e.g., removing long branches and/or bootstrapping single-gene trees prior to concatenation), and easily replace the Hook Database with a set of gene families of interest; these features extend beyond previous phylogenomic approaches (e.g., references 2, 5, 6). In addition to varying the input data, EukPhylo users have a large amount of leeway in deciding how to remove putative contamination from their data (e.g., by setting rules for sister/subsister with or without branch length constraints, and exploring different numbers of taxa in parameterizing “clade grabbing”). Furthermore, as we demonstrate in our EGT analyses, EukPhylo’s suite of stand-alone utility tools allows users to explore hypotheses relevant to their particular questions.

Our exemplary analysis of 500 conserved gene families demonstrates the power of EukPhylo to analyze large, diverse eukaryotic data sets, and to improve topologies through tree-based contamination removal. Even the first species trees produced by EukPhylo from both the concatenated and Asteroid analyses prior to phylogeny-informed contamination removal are largely concordant with published literature (Fig. 4a; Table S12 at Figshare), particularly for morphologically defined clades like dinoflagellates, animals, red algae, Tubulinea, and Euglenozoa (e.g., references 1, 2, 46–48). Importantly, many previously published analyses of EToL rely on far fewer genes and taxa, and some fail to demonstrate monophyly of clades with robust synapomorphies.

The EukPhylo phylogeny-informed contamination loop improves estimates of EToL by removing putative contaminants, first based on sister/subsister analysis (Fig. 4b) and then by retaining sequences for which we have the greatest confidence through “clade grabbing” (Fig. 4c). Following these steps, we see additional major clades supported (e.g., Opisthokonta, Alveolata). Importantly, we recover SAR and the sister relationship between the genus *Rhodelphia* and red algae only after removal of gene families most affected by putative EGT (Fig. 4d); these analyses suggest that EGT may be a driver in inferences about EToL. Finally, we do not recover a number of eukaryotic “supergroups” like Amorphea, CrumS, Cryptista, Diaphoretickes, Haptista, Obazoa, or TSAR (Fig. 4; Table S12 at Figshare), suggesting the possibility that they emerged through resampling of the same data across analyses.

Across all stages of the contamination loop, we obtain a root among excavate taxa (e.g., parabasalids, fornicate, both of which were formerly assigned to the “supergroup” Excavata), generally consistent with the hypothesis in Al Jewari and Baldauf (46). However, the placement of these lineages plus a few orphan species at the root of EToL may be due in part to a high amount of missing data; clades with the greatest proportion of gaps and fewest numbers of gene families (e.g., Breviata, Fornicata, Jakobida, Malawimonadida, Microsporidia, and Preaxostyla) tend to be most unstable across analyses and to fall close to the root of the eukaryotic portion of the tree (Fig. 4; Table S14 at Figshare; Fig. S4). Alternatively, the long branches of these predominantly parasitic lineages may drive the placement of these lineages toward the root of EToL; rigorously testing the root would likely require more attention to gene-family selection, visual inspection of individual gene trees, as well as mitigation of the effect of both missing data and long branch attraction.

In sum, EukPhylo allows for “phylogeny on the fly” as users can reset gene families and contamination removal rules, and then run the pipeline and associated toolkit with flexibility, modularity, and transparency. EukPhylo can also allow researchers to rapidly compare hypotheses regarding the placement of disputed lineages (e.g., Telonemia [48] or Hemimastigophora [14]) through taxon-rich analyses and by leveraging the ability of the contamination loop to treat data from “orphan” taxa differently (e.g., more leniency in curation) than data from taxa belonging to better sampled clades. Moreover, because researchers can choose gene families independently for each study for as many as 1,000 taxa as done in this study (or by using a custom-built “hook”), EukPhylo will help to mitigate the problem of recovering similar topologies across resampled data sets (e.g., references 15, 45, 48, 49). In sum, EukPhylo provides a broad set of tools to facilitate large phylogenomic analyses from start to finish, providing a model for establishing best practices in a field that now relies on ‘omics data from diverse lineages.

### Caveats

There are several important caveats to consider when using EukPhylo. While the EukPhylo pipeline is built to be generalizable, it includes stringent data-quality filters that may remove sequences of interest in certain studies (i.e., false negatives) and is therefore best suited for processing data for large-scale evolutionary or population-level analyses (e.g., generating many diverse gene trees for a supertree approach to phylogeny). Given this, EukPhylo is likely not an appropriate tool for the study of individual gene families, where more nuanced curation is required to interpret gene loss, lateral gene transfer, and the placement of fast-evolving sequences. Hence, users interested in the evolutionary history of specific genes should use EukPhylo with caution as vertically inherited sequences may be removed by quality filters and through the contamination loop. To mitigate this, EukPhylo makes it easy to detect cases where “good” sequences are removed by the contamination loop as it provides intermediate files and lists of removed sequences as output for inspection by users.

More broadly, parameters that we applied universally (such as the Guidance sequence cutoff, [52, 53]) are likely not appropriate for all taxonomic groups, and there is room for improving the flexibility of parameter fitting for each taxon. An alternative approach would be to inspect per-sequence Guidance scores for every gene of interest, resetting cutoffs depending on score distributions (i.e., an approach analogous to the use of a gamma parameter to model rate heterogeneity in phylogenetics). Finally, we note that though the stochasticity associated with aligning sequences and building gene trees makes some aspects of analyses not completely replicable, the structure of EukPhylo increases transparency (i.e., by recording user-defined rules and removed sequences) to enable streamlined and large-scale phylogenomic studies.

### Synthesis

Currently, studies of microbial eukaryotes rely heavily on bioinformatics tools developed for macrobes and/or bacteria; however, such tools do not incorporate workflows that are critical for accurate analysis of eukaryotic lineages, where the underlying data must be rigorously cleaned in light of contamination and non-vertical gene transfer (i.e., LGT and EGT). In light of increased attention to the importance of democratizing biology research, especially in the realm of software tools (71–73), we designed EukPhylo to be accessible to researchers with a limited bioinformatic background. Combining the novel phylogeny-informed contamination removal methodology with the modularity that enables users to integrate their preferred phylogenetic/phylogenomic approaches, EukPhylo has the potential to improve standards and increase the repeatability of studies of eukaryotic phylogeny.

Our intention with the case study of 500 gene families across 1,000 species is to demonstrate the flexibility and power of EukPhylo, setting the stage for other researchers to deploy EukPhylo to assess hypotheses on EToL, and on eukaryotes in general. For example, an assessment of the root of EToL could be done by using EukPhylo tools to simultaneously select gene families that likely originated in LACA (the last common ancestor of eukaryotes and archaea, representing the host at eukaryogenesis) and in LBCA (genes that may trace to a common ancestor of eukaryotes and bacteria, a set that would include contributions from the ancestral mitochondrion plus other “ghost” bacterial symbionts). Scientists interested in gene family evolution can either add in their own reference database for GF assignment or select from our ~15,000 gene families to explore gene sets underlying the systems such as the cytoskeleton, metabolism, central dogma, and much more as demonstrated by our analysis of an epigenetic toolkit (74); however, those interested in this type of approach should carefully read the caveats section above. Because of its modularity, EukPhylo outputs can be used alongside other phylogenomic/bioinformatic tools to allow users to deploy a plurality of approaches in analyzing data, including in identifying orthologs and supporting the generation of multiple sequence alignments for analyses of structure with tools like AlphaFold (75). In sum, we are optimistic that EukPhylo will enhance exploration of gene and genome evolution in diverse eukaryotes.

## MATERIALS AND METHODS

Here, we provide an overview of methods, including descriptions of taxa and gene families, the development of the EukPhylo Hook Database, brief descriptions of the functionality of EukPhylo, and details on our exemplary analyses of 500 gene families in 1,000 taxa. Further details are provided in the supplemental text section within the Supporting Information.

EukPhylo v.1.0 is based on carefully controlled names of both clades and species that facilitate analyses. Each transcriptome and genome in the EukPhylo database is identified using a 10-digit code, which represents either an individual cell or GenBank accession, or a pool of transcripts as noted in Table S1 at https://figshare.com/projects/EukPhylo_Supplemental_Files/196552. The first two digits of the code identify one of eight “major” clades as follows: Ba, Bacteria; Za, Archaea; Op, Opisthokonta; Am, Amoebozoa; Ex, excavate lineages (formerly the clade “Excavata”); Sr, SAR (Stramenopila, Alveolata, and Rhizaria); Pl, Archaeplastida; EE, orphan lineages. The next two digits identify the taxonomy of the taxon at the “minor” clade level (e.g., within Opisthokonta are the minor clades Op_me for metazoa; Op_fu for fungi; Op_ch for choanoflagellates; and Op_ic for Ichthyosporea; Table S6 at Figshare). The last four digits identify the species and, if applicable, sample ID within a species (e.g., Am_tu indicates the minor clade Tubulinea, and there are multiple samples of *Hyalosphenia papilio*, identified as Am_tu_Hp01, Am_tu_Hp02, etc.; Table S1 at Figshare). GFs are identified as per the notation in OrthoMCL version 6.13 (54), with the prefix OG6_ followed by a unique six-digit sequence (see sections on Hook Database and composition-based curation below). All sequence identifiers used in EukPhylo databases are unique and begin with the 10-digit taxon identifier, then are labeled by a unique contig/CDS ID designated either by an assembler or by annotations as downloaded from GenBank, and end with a 10-digit GF identifier.

### Development of the Hook Database

As a starting place for evolutionary analyses of lineages sampled across the EToL, we developed a Hook Database of 15,138 GFs selected for presence across a representative set of eukaryotes. The Hook allows assignment of sequences to GFs and can easily be replaced by researchers interested in specific gene families (e.g., gene families involved in epigenetics, in meiosis, etc.). To develop the Hook Database (Fig. S1), we started with “core” orthologs from the OrthoMCL version 6.13 database (495,339 GFs). We then proceeded to several curation steps to achieve the following goals of (i) reducing the database size while retaining diversity within eukaryotes; (ii) retaining only GFs that are present in a representative set of eukaryotes given our focus on microbial lineages (i.e., we undersample animal-specific and plant-specific GFs); and (iii) removing GFs and sequences within GFs that are likely to cause sequences to be misassigned or assigned to groups of sequences without useful functional meaning (e.g., sequences that comprise only a single common domain or chimeric sequences). To accomplish these goals, we assessed the taxonomic diversity and the quality of each GF using a variety of custom scripts (DOI:10.5281/zenodo.13323372). We detail these curation steps in the Ssupplemental Material and reiterate the goal of generating a set of representative gene families to use in analyses of diverse eukaryotes.

#### EukPhylo part 1

EukPhylo comprises two components: the first (EukPhylo part 1) provides initial gene family assignment to sequences and the second (EukPhylo part 2) builds alignments and phylogenetic trees. Central to all of EukPhylo is the use of consistent taxon codes (see above). EukPhylo part 1 has two versions. The first is intended for use with transcriptomic data and accepts as input assembled transcripts as produced by rnaSPAdes (Fig. S2 at https://figshare.com/projects/EukPhylo_Supplemental_Files/196552). Users may use other assembly tools, as long as sequence names follow the rnaSPAdes output format (i.e., including a contig identifier, k-mer coverage, and length). The second version is for use with whole genome data and accepts as input nucleotide CDSs. Each step can be run individually across any number of input samples; runs can be paused and resumed at any stage, and this can be flexibly managed using a wrapper script provided in the Zenodo repository (DOI:10.5281/zenodo.13323372).

##### Transcriptomic pipeline

The transcriptomic pipeline requires three inputs: a fasta file of correctly named contigs (see manual), a file specifying a genetic code (if known) for each taxon, and for those interested in removing sequences misidentified due to index hopping, a list of names of conspecifics (i.e., taxa/samples that are expected to share identical nucleotide sequences). As described in detail in the Supplemental text and in Fig. S2, EukPhylo part 1 removes sequences based on length parameters, and optionally, sequences that are likely incorrectly labeled due to index hopping (76, 77) in the same sequencing run. Next, putative rRNA sequences are moved to a separate folder, and remaining sequences are labeled as possible prokaryotic contamination (ending in _P) for users to inspect downstream. To provide initial gene family assignments, Diamond (54) is used to compare sequences either to the EukPhylo Hook Database (described above) or a user-provided database. As the Hook Database is replaceable and customizable, this step offers an opportunity to filter transcriptomic data for a group of gene families/functional groups of interest. EukPhylo then captures open reading frames (ORFs) as both nucleotide and amino acid sequences. Finally, EukPhylo part 1 removes putative chimeric and partial transcripts to produce “ReadyToGo” fasta files and calculates various statistics for both sequences and taxa.

##### Genomic pipeline

The version of EukPhylo part 1 applicable to coding domain sequences from whole genome assemblies is similar to the version for transcriptomic data described above and in the Supplementary Text, but with some important differences. Given that coding domains are already determined, this version of EukPhylo part 1 has no length filter and instead immediately evaluates in-frame stop codon usage and translates the nucleotide CDSs to amino acids, at which point it uses Diamond BLASTp to assign gene families against the same reference database (in our analyses, the Hook Database). Next, the pipeline filters sequences by relative length, removing any sequence less than one-third or more than 1.5 times the average length of its gene family in the Hook Database. After some reformatting, EukPhylo part 1 then outputs the same “ReadyToGo” files as the transcriptome version of the pipeline: a nucleotide and amino acid fasta file with gene families assigned for each taxon, a tab-separated file of BLASTp data against the Hook Database, and summary statistics.

### EukPhylo part two

The second major component of the pipeline (EukPhylo part 2) starts from the “ReadyToGo” files produced by part 1 (or any set of per-taxon sequences with names that match PLT6 criteria) and generates multisequence alignments and trees. Prior to running Guidance (52, 53) for homology assessment, optional filters are available in the script “preguidance.py,” to select the sequences to use for the analysis based on GC composition or high similarity proportions (details in the Supplemental text), on the whole data set or on specific taxon.

Then EukPhylo part 2 runs Guidance version 2.0.2 (52, 53) in an iterative fashion to remove non-homologous sequences defined as those that fall below the sequence score cutoff. (We note that there is some stochasticity here given the iteration of alignments built into the method.) After inspecting a diversity of gene families, we have lowered the default sequence score cutoff from 0.6 to 0.3, though this may not be appropriate for all genes (see caveats section below). To remove regions with large gaps that can confound tree building, the resulting MSAs are then run through TrimAl (78) to remove all sites in the alignment that are at least 95% gaps (again, a parameter a user could alter). The last step of EukPhylo part 2 before phylogeny-based contamination removal is to construct gene trees, though users can stop EukPhylo after Guidance to build trees with other software as they prefer. Currently, EukPhylo supports RAxML (79), IQ-Tree (with the hardcoded protein LG+G model [59]), and FastTree (80).

#### Phylogeny-based contamination removal

A key innovation in EukPhylo v.1.0 is the “contamination loop,” an iterative tool to identify and remove contamination based on analyses of single-gene trees. This tool incorporates two main methods of contamination assessment informed by tree topology. The first method—“sisters” mode—is intended to target specific instances of contamination. It enables users to remove sequences based on cases of repeated contamination in target taxa, determined by prior assessment of trees (aided by the utility script ContaminationBySisters.py or known contaminants; Fig. 3). We provide additional details in the Supporting Information. The second method—“clade-based contamination removal”—is intended for cases when the user is interested in genes present in a group of organisms with multiple representative samples and/or species in the gene trees (Fig. 3). For a given set of target taxa, this method identifies monophyletic clades containing those taxa within each gene tree (allowing a user-set number of contaminants) and re-aligns and re-builds the tree excluding all sequences from the target taxa that do not fall into these robust clades. In both cases, sister and clade grabbing, a user-defined set of rules is necessary and can be built using the set of utility scripts provided with the main pipeline. Given that these methods incorporate tree building on each iteration, users should expect some amount of stochasticity in which sequences are removed.

#### Ortholog selection for concatenation

EukPhylo part 2 includes an option to concatenate representative sequences per GF into a supermatrix from which users can construct a species tree. This can be done as part of an end-to-end EukPhylo run, or by inputting already complete alignments and gene trees and running only the concatenation step. If a GF has more than one sequence from a taxon, EukPhylo keeps only the sequences falling in the monophyletic clade in the tree that contains the greatest number of species of the taxon’s clade as determined by its sample identifier. If multiple sequences from the taxon fall into this largest clade, then the sequence with the highest “score” (defined as length times k-mer coverage for transcriptomic data with k-mer coverage in the sequence ID as formatted by rnaSpades, and otherwise just length) is kept for the concatenated alignment. If a GF is not present for a taxon, its missing data are filled in with gaps in the concatenated alignment. Along with the concatenated alignment, this part of the pipeline outputs individual alignments with orthologs selected (and re-aligned with MAFFT), in case a user wants to construct a model-partitioned or other specialized kind of species tree.

### Analysis of 500 GFs from 1,000 species

To demonstrate the power of EukPhylo, we conducted a phylogenetic analysis on 500 conserved gene families among 1,000 species (Fig. 4). Selection of taxa and gene families to include in this study was based on quality of data and taxon presence. We went through several rounds of curation and selection that are detailed in the Supplemental text; the final selection of taxa is described in Table S1 and S7 at https://figshare.com/projects/EukPhylo_Supplemental_Files/196552. We used EukPhylo part 1 to produce fasta-formatted CDS files (genomes) and assembled transcripts (transcriptomes) for each of the genomes and transcriptomes downloaded from public databases plus data generated in our lab.

We then reran EukPhylo part 2 with these 1,000 taxa, using only the sequences labeled as “OG6,” based on GC composition (see Supplemental Material for details), with five iterations of Guidance (52, 53), and built trees using IQ-Tree (-m LG+G; File S4 at Figshare). For this study, we also implemented the “similarity filter” with an amino acid identity cutoff of 99% to remove highly similar sequences within species (see Supplemental Methods). We then removed sequences identified as contaminants by the contamination loop in EukPhylo part 2. We first ran 10 iterations in “sisters” mode, using the rules file provided in Table S8 at Figshare, followed by 5 iterations of “subsisters” rules on a select number of taxa (Table S9 at Figshare). Next, we ran two separate iterations of the “clade” mode, the first one to remove only the ciliate parasites of Parabasalids (Ex_pa) that occurred when transcriptome data were generated from co-contaminated rumen ciliates, and the second one to remove sequences from all other well-sampled taxa (see Table S10 and S11 at Figshare for rules and Supplemental material for details).

For the final analyses, we removed gene families that showed evidence of either primary or secondary EGT. We first used the utility script CladeSizes.py to identify trees where multiple photosynthetic lineages nest in a single clade. We identified putative primary EGT events as clades comprising only photosynthetic eukaryotes and bacteria (and occasionally archaea), with many of these including cyanobacteria; we used this broad approach in light of the possibility of either LGTs among prokaryotes (i.e., from cyanobacteria to other prokaryotes) after transfer to eukaryotes, and because of the possibility of multiple sources of photosynthetic machinery in eukaryotes (e.g., reference 81). We identified putative secondary (or tertiary) EGT events as cases in which we found interdigitation of multiple lineages of photosynthetic eukaryotes (e.g., photosynthetic stramenopiles nested in red algae). We manually examined all trees with a large number of putative primary and/or secondary EGT events (identified using the utility script CladeSize.py), resulting in a set of 169 OGs total that we removed to construct our final EGT-removed species tree (Fig. 4d).

To build species trees, we used two methods: Asteroid (61) and the concatenation option included in EukPhylo part 2. For concatenation, we selected orthologs (i.e., removed putative paralogs) at each stage and built a concatenated alignment (see Supplemental material and the EukPhylo v.1.0 GitHub wiki page for more information); species trees were then built with IQ-Tree (-m LG+G; File S4 through S6 at Figshare). We also used Asteroid (61) to build supertrees using single-gene trees generated by EukPhylo, at each step of the contamination loop (File S9 at Figshare). We ran this conserved GF analysis with Guidance v.2.0.2 as this was the version available at the time, but we subsequently updated the pipeline and estimated the performance with Guidance v.2.1 accessed in June 2024 (Table S5 at Figshare).

## Data Availability

The main EukPhylo pipeline and accompanying scripts, including all scripts used for this study, are available on GitHub (https://github.com/Katzlab/EukPhylo) and Zenodo (DOI:10.5281/zenodo.13323372). All results and outputs generated by this study, including Tables S1 to S15 and Files S1 to S10 listed in the article, are available on Figshare (https://figshare.com/projects/EukPhylo_Supplemental_Files/196552).

## SUPPLEMENTAL MATERIAL

Supplemental Material mbio.01770-25-s0001.pdf Supplemental text and figures.

ASM does not own the copyrights to Supplemental Material that may be linked to, or accessed through, an article. The authors have granted ASM a non-exclusive, world-wide license to publish the Supplemental Material files. Please contact the corresponding author directly for reuse.
