# Supplementary material for: Rethinking large-scale phylogenomics with EukPhylo v.1.0, a flexible toolkit to enable phylogeny-informed data curation and analyses of diverse eukaryotic lineages
Source: mBio. 2025 Aug 27;16(10):e01770-25. doi: 10.1128/mbio.01770-25 (PMC12506077; doi:10.1128/mbio.01770-25)
Supplement: Supplemental Material — Supplemental text and figures. [file mbio.01770-25-s0001.pdf]

Supporting text and figures for
Rethinking large scale phylogenomics with EukPhylo, a flexible toolkit to enable phylogeny-informed data curation and analyses of diverse eukaryotic lineages.

Laura A. Katz, Auden Cote-L'Heureux, Marie Leleu, Godwin Ani, Rebecca Gawron

Laura A. Katz
Email: lkatz@smith.edu

This PDF file includes:

Supplemental text
Figures S1 to S4
Supplemental References

Other Supporting Materials for this manuscript, include the following, and be found here: https://figshare.com/projects/Rethinking_large_scale_phylogenomics_with_EukPhylo_a_flexible_toolkit_to_enable_phylogeny-informed_data_curation_and_analyses_of_eukaryotic_genomes/196552

Supplemental Text: Details on Methods

Development of the Hook Database

The Hook Database went through multiple rounds of refinement as we worked to generate a representative set of 10,000-20,000 gene families for analyses of diverse eukaryotes (Figure S1). Starting with 'core' orthologs from the OrthoMCL version 6.13 database (495,339 GFs), the first filter removed GFs with fewer than 25 species and an average sequence length of less than 100 or more than 5000 amino acids. We then removed any sequence <50% or >150% the average length of the GF (as defined by OrthoMCL) as long as at least one representative of that sequence's minor clade remains (see Table S6 in Figshare for details of minor clades). At this point, to reduce the size of the database and number of redundant sequences, we used CD-Hit to cluster sequences within each minor clade (Table S6) within each GF. We clustered sequences at 95% amino acid identity over at least 67% of the smaller sequence, keeping the longest sequence from each cluster as the representative. By this method, each GF retained at least one representative from each minor clade previously present in that GF. After this stage, 398,157 GFs remained.

Filtering GFs based on taxonomic presence. With the goal of capturing GFs that would be of use to the community studying microbial eukaryotic species, we focused our next effort on smaller GFs that were present in lineages that had only a few closely-related representatives in the OrthoMCL database (e.g. ciliates and cryptophytes). We compiled a list of these taxa (called 'BLAST-able taxa'; Table S15 in Figshare) for use in the following steps. The purpose here was to reduce the set of reference GFs to those most useful for phylogenetic analysis by keeping GFs present across multiple widely-sampled lineages and GFs that are seemingly present only in poorly-sampled lineages because of the taxonomic representation in OrthoMCLs.

With the aim of further refining the Hook DB, we classified GFs considering their taxonomic distribution: we described 4 categories: G1NS ("Greater than 1, No Singletons"; GFs present in at least two minor clades of at least 2 major clades); G1HS ("Greater than 1, High Singletons"; GFs present in at least two major clades, but in only one minor clade in all or all but one of the major clades), 1MCG1mc ("1 Major Clade Greater than 1 minor Calde"; GFs present in only one major clade, but more than one minor clade); and 1mc1mc ("1 Major clade, 1 minor clade"; GFs present in only one minor clade). We only retained GFs in the 1MCG1mc and 1mc1mc categories if a representative of a BLAST-able taxon (Table S15 in Figshare) in the GF had a significant BLAST hit against a good diversity of genomes, using a custom reference database (File S10 in Figshare). Here, we retained a GF if a sequence from a BLAST-able taxon in that GF hit at least 80% of the genomes in its major clade, as long as there were more than 10 genomes from the major clade of the BLAST-able taxon in the dataset of diverse genomes. Otherwise, the sequence had to hit at least 50% of genomes. We took extra care in capturing GFs for several minor clades that are poorly represented in OrthoMCL; we retained BLAST-able taxon sequences from one of the groups Amoebozoa (Am), Fornicata (Ex_fo), ciliates (Sr_ci), and Stramenopila (Sr_st) that hit at least 66% of the members of that group present in the set of diverse genomes; GFs with Fornicata and Heterolobosea sequences could also be retained if

they hit at least 66% of excavate genomes, and chromerids if they hit at least 66% of apicomplexan genomes. GFs with any non-metazoan or non-fungal sequences were retained if these sequences hit at least 50% of either metazoan or fungal genomes.

Removing sequences - homology assessment. Following these criteria, GFs from all four categories containing at least four sequences were then iteratively run through Guidance v2.0.2 (Sela et al., 2015) to remove non-homologous sequences. At each round all sequences with a score less than 0.3 were removed until no sequences with a score less than 0.3 remained. GFs in the G1NS and G1HS categories that were reduced in size by Guidance and therefore switched to the 1MCG1mc or 1mc1mc categories were retained only if they contained BLAST-able taxa that met the BLASTp criteria above. GFs with fewer than 4 sequences were retained but not run through Guidance.

Removing sequences - chimeras and pseudogenes. Next, to ensure the removal of chimeric sequences, pseudogenes, and those with regions that may have spuriously attracted sequences to GFs to which they do not belong (i.e. sequences with mis-annotated introns as insertions), we removed individual sequences that had contiguous gaps longer than 25 amino acids. An exception was made for cases where these sequences were from the list of poorly represented taxa (Table S15 in Figshare) as these gaps might represent novel proteins rather than annotation errors. Sequences from these 'blastable' taxa were removed only if the GF included at least one other 'paralog' from the taxon without the inserted region; otherwise, for these poorly-represented taxa, the gap length maximum was increased to 50 amino acids to account for divergence within taxa that lack close relatives.

Removing redundant GFs. To reduce the arbitrary assignment of sequences to one of multiple GFs with very similar sequences, we used Diamond to identify GFs that could be considered 'oversplit' (i.e. they may belong to a larger GF). We used BLASTp to similarity-search the Hook database against itself and then removed all small GFs (defined as those present in fewer than six sequences or only a single minor clade) with at least one sequence if they hit a sequence from another GF with an e-value less than 10e-25. In the case where the other GF was also small, we kept the larger of the two. If both GFs were small and had the same number of sequences, we kept the one with longer average length. Then we applied an additional sequence-level filter for GFs with an average length greater than 300 amino acids. If the GF had 10 through 20 sequences, we removed any sequence in the GF that hit a sequence from another GF if it was shorter than the 10th percentile of length among sequences in the GF or longer than the 90th percentile, as long as at least one member of the minor clade of a removed sequence remained. If the OG had more than 20 sequences, we removed any sequence in the GF that hit a sequence from another GF if it was shorter than the 5th percentile of length in the GF or longer than the 95th percentile, as long as at least one member of the minor clade of a sequence removed remains.

Removing GF based on taxonomy and trimming sequences. At this stage, we manually assessed the taxonomic presence of each GF. Given our focus on diverse microeukaryotes, we removed GFs present in only Metazoa (Op_me), Fungi (Op_fu), and Chlorophyta (Pl_gr), and

GFs with only one sequence as long as that sequence was not from a poorly-represented taxon. To further reduce the prevalence of chimeric sequences, we trimmed GFs with an alignment length greater than 300 amino acids and that contain at least 20 sequences to capture the best estimate of start and stop locations: if the OG had a methionine shared by 30% of sequences in the first 20% of the alignment, we removed everything ahead of the methionine; we also removed all sequences in the alignment after the point in the alignment that 90% of sequences had ended. In revising the manuscript, we also realized that we had inadvertently corrupted the files of 276 OGs by retaining only the first 60AA of these gene families, so we removed. The final Hook Database can be found in File S8 in Figshare.

Replacing the Hook database. Users can easily replace the Hook database with genes of interest, a feature of the module nature of EukPhylo. To do so, it's necessary for the new database to match a few simple criteria: 1) sequences must be formatted with the same nomenclature as the Hook or ReadyToGo files (a 10 digit code specific for each species, followed by a unique identifier if needed and the GF name; see the main text for more details); 2) all sequences, for all GFs, must be in a single fasta file, and 3) users must create a Diamond database out of this file and place it in the Database folder when running EukPhylo part 1.

Functional analysis of the Hook Database. To determine the function of GFs in the Hook database, we used EGGNOG-mapper version 2.0 (Cantalapiedra et al., 2021) to annotate the Hook amino acid sequences based on orthology relationships to the EGGNOG 5.0 reference database (Huerta-Cepas et al., 2019); this returned GO, PFam, EC, COG, and KEGG terms for sequences in which an orthologous relationship could be determined. We expanded the set of GO terms for each sequence by converting PFAM domains identified by EGGNOG-mapper to GO terms with Goatools (Klopfenstein et al., 2018) and custom Python scripts, and we provide summarized GOs per OG (Table S4 in Figshare).

EukPhylo Part 1 – Transcriptomic pipeline: an in-depth description

Input samples require three starting files: a fasta file of correctly named contigs, a file specifying a genetic code (if known) for each taxon, and for those interested in removing sequences misidentified due to index-hopping, a list of names of conspecifics. A set of 10 transcriptomes, with all outputs produced by EukPhylo part 1, is available at https://github.com/Katzlab/EukPhylo-6/tree/main/PTL1. First, excessively short or long assembled transcripts are removed, with default cutoffs of 200 bp and 15kb (1a_TranscriptLengthFilter.py). Next is an optional step (1b_CrossPlateContamination.py) to remove contaminating sequences introduced by index hopping (Costello et al., 2018; MacConaill et al., 2018), which are defined as sequences with lower k-mer coverage in the contaminated taxon than the contaminating taxon. We recommend applying this "cross plate contamination" (XPC) step to sets of samples processed and/or sequenced together (e.g. all samples from the same sequencing plate). This XPC step clusters sequences across all input samples at a percent nucleotide identity of 99%, and then removes sequences with less than one tenth the k-mer coverage (as calculated by rnaSpades) of the highest covered sequence in the cluster, as long as both sequences are not from the same taxon. With the default

parameters, no sequence with a k-mer coverage greater than 50 is removed, and users can adjust this in the base scripts if they choose to, or they can skip this step altogether.

In the next steps of EukPhylo part 1, several custom databases are used as references to remove rRNA and to label sequences as likely prokaryotic contamination (appending _P to sequence names), and all information is recorded for users to inspect downstream. These databases can be found on Figshare here: https://figshare.com/articles/dataset/PhyloToL_part_1_Databases/26597368?file=48424516. Ribosomal RNA is identified (2a_Identify_rRNA.py) using nucleotide similarity search (BLASTn), and sequences that hit the diverse reference database ("SSULSUdb") with an e-value less than 1e-10 are removed to a separate output file. Sequences that similarity search (Diamond BLASTp) to an amino acid prokaryotic reference database ("micout") with an e-value more than 100 times that of their hit to a similar eukaryotic reference database ("eukout") are labeled with a "P" in the sequence name as possibly of prokaryotic origin and either recent gene transfers or contamination (2b_Identify_Proks.py). Sequences with an e-value against the eukaryotic database more than 1000 times that of the e-value against the prokaryotic database are labeled with an "E" in the sequence name. All other sequences are labeled with "U" for "unknown." All of these labels are carried forward through alignments and trees, giving users additional information as they inspect outputs.

To provide initial gene family assignments, sequences are then compared either to the EukPhylo Hook Database (described above) or a user-provided Diamond-formatted database for GF assignment. To create this customized reference database, users should create a fasta file containing amino-acid sequences from a representative set of gene families in a representative set of taxa given the research question. For example, if studying genes related to meiosis in foraminifera (where gene function assignments are lacking), a user might create a set of meiosis-related genes from a wide range of taxa. In this case, EukPhylo part 1 would output "ReadyToGo" files (see below) of only sequences roughly homologous to this set of meiosis genes, which users can use for downstream analyses in EukPhylo part 2. The sequence IDs in the reference .fasta file should all begin with a ten-digit taxon identifier and end with a 10-digit OG identifier as used in EukPhylo (i.e., OGx_xxxxxx, following the pattern of OG identifiers in OrthoMCL). Using Diamond BLASTx similarity search, sequences are then assigned to a GF if their best hit against this reference database returns an e-value lower than e-15 (3_AssignOGs.py). Sequences not assigned to an GF are not carried forward in output files past this stage.

EukPhylo is flexible in its ability to work with data from across the microeukaryotic tree of life, including from species with stop codon reassignments (e.g. many ciliates). Users can input genetic codes from a selection (Universal, *Blepharisma*, *Condylostoma, Chilodonella, Euplotes, Peritrich, Vorticella,* and "none") at the start of the EukPhylo part 1 run either by giving a single choice for all taxa or by inputting a file that assigns a genetic code individually to each sample. However, for users wishing to explore genetic codes, EukPhylo part 1 reports in-frame stop codon frequencies. This is done by translating all sequences with each stop codon individually and assessing the in-frame frequency of the other two stop codons

(4_InFrameStopCodonEstimator.py). Users then need to evaluate this output (gcode_output.tsv): if a stop codon appears in-frame at a relatively high frequency when sequences are translated using the other two stop codons as STOP, then it may be that the codon has been re-assigned. EukPhylo part 1 does not determine the amino acid to which the stop codon has been reassigned, as this is a complex issue best determined by in-depth analysis of multi-sequence alignments.

Next, sequences are translated using the DIAMOND BLASTp results from GF assignment as a starting point for determining coding sequence boundaries (5_GCodeTranslate.py). The first in-frame start codon (if the 5' boundary of the DIAMOND hit is not at a start codon) and last in-frame stop codon (using the assigned genetic code) outside of these bounds are found, while ensuring that in-frame stop codons are not introduced (given the nature of transcriptomic data, poor genetic code assignment or low-quality/partial data can interfere with this process). Otherwise, the bounds of the best DIAMOND hit are used. From this point forward, nucleotide and amino acid data are considered separately, but any nucleotide sequence removed in downstream curation is also removed from the amino acid sequence set and *vice versa*.

Lastly, two filters are applied (6_FilterPartials.py) to remove low quality chimeric or partial transcripts. First, all sequences shorter than 33% or longer than 150% the average length of sequences from the same GF in the Hook database are removed. Then, for each transcriptomic sample, all sequences within an GF are compared at the nucleotide level to the sequence with the highest "score" (defined as k-mer coverage multiplied by length). Any sequence greater than 98% identical over 20% of its length to this highest scoring sequence is removed. The sequences and files are then reorganized into "ReadyToGo" files (Table S1 in Figshare), which are fasta files which contain the final set of curated sequences with GFs assigned by the pipeline (both nucleotide and amino acid).

EukPhylo also has tools that generate spreadsheets for per-sequence as well as per-taxon analyses by users. The pipeline produces a tab-separated spreadsheet of BLASTx hit data from the gene-family assignment against the Hook Database and several summary output files describing sequence composition. These include a file of aggregate statistics per taxon (e.g. number of sequences assigned GFs, number of GFs, average GC content at silent sites, and more) as well as a file for each taxon with sequence-level statistics (length, GC content, expression, etc.).

EukPhylo Part 2 – Pre-Guidance processing
After EukPhylo Part 1, users can run optional filters in generating "Pre-Guidance" files, which are required to move to alignment and tree building stages. Pre-Guidance files takes as input ReadyToGo-formatted files (one file per taxon with amino acid sequences, sequence IDs starting with a ten digit taxon identifier and ending with a ten digit GF identifier) and outputs non-aligned fasta files for each GF.

GC content assessment. One filter aims to remove sequences outside silent-site GC content ranges set by the user, and relies on the output of the utility script 'GC_Identifier.py.' To use this

script, users need to assign compositional bounds (upper and lower values of silent-site GC content) after running the codon analysis utility script (CUB.py) on outputs from EukPhylo part 1. Then this utility script renames GFs in ReadyToGo files with the prefix ‘OGA’ if the sequence is below the lower compositional bound (AT rich), ‘OG6’ if between the lower and upper bounds, and ‘OGG’ (GC rich) if above the upper compositional bound. When running EukPhylo part 2, a user can input a specific OG prefix (OGA, OG6, and OGG) and the alignments/trees will be built using only sequences with that prefix. If no OG prefix is input, all sequences will be taken. Hence, a user might choose to only use sequences with the prefix OG6 if they are confident that this (blunt) approach will remove contaminants, or they can keep all sequences and then inspect by prefix on resulting trees.

In our case study, we used a hybrid approach to filtration by composition at this stage. To inspect the quality of both transcriptomes and genomes, we plotted each sequence on a composition plot as described in our methods. As each taxon has a specific fingerprint on these plots, we were able to identify and label the sequences we considered as likely contaminants as they fell outside of the taxon-specific GC composition range (File S3 in Figshare). The quality of data varies among samples, going from the cleanest (i.e. a small range of silent site GC content) to showing the presence of a whole community of species (a large range). For this analysis, we moved forward with only the sequences falling inside the ranges of GC composition; our chosen bounds can be found in Table S1 in Figshare.

Similarity filter. The second optional filter in the ‘pre-Guidance’ step of EukPhylo part 2 allows users to collapse closely-related sequences within taxa, which might represent ingroup paralogs, redundant transcripts or alleles. For each taxon, all sequences in each GF are similarity-searched against the longest sequence using Diamond, and sequences that match to this longest sequence with a percent identity greater than a user-input cutoff (usually very high, e.g. 99%) are removed. If users are concerned about only a particular taxon or set of taxa having highly redundant sequences (e.g. human genome), or want to remove highly similar sequences from all but a focal group of taxa, they can input a list of taxa on which the similarity filter is to be exclusively applied. Hence, ingroup paralogs can be retained from focal lineages and removed from outgroups.

EukPhylo Part 2 – Contamination loop details

Another unique feature of EukPhylo is the phylogeny-informed contamination removal, which is repeatable and transparent to others where users can publish associated rules and lists of removed sequences.

Sisters removal. In ‘sisters’ mode, any sequence found sister to a known contaminant will be removed using a set of user-defined rules. Though in some cases sources of contamination are obvious (e.g. food sources, known symbionts), care must be exercised with using this option, and particularly for taxa of unknown phylogenetic position; in our example study (see main methods), we provide a list of rules deployed, enabling users to explore parameters and to repeat analyses. To make sister-based contamination removal more conservative, users can input a maximum branch length for a sequence to be removed relative to the average (e.g. a

sequence might only be removed if sister to a known contaminant with a branch length less than 0.5 the average node-to-tip in the tree). We also provide a 'subsister' option to cover instances where two closely related samples are co-contaminated, so each taxon only appears sister to the other, but the node parent to both taxa might be sister to the common contaminant. In other words, subsister mode specifies that a sequence should be removed if its parent node is sister to a known contaminant. Given that these methods incorporate tree-building on each iteration, users should expect some amount of stochasticity in which sequences are removed; hence we recommend running this mode through five or more iterations.

Clade Grabbing. Clade-based contamination removal is intended for cases when the user is interested in retaining genes with the greatest confidence based on the numbers of species in clades (Fig. 3). To help in establishing criteria for clade-grabbing, we provide a utility script, CladeSizes.py, that generates a spreadsheet describing the distribution of monophyletic clade sizes for each taxonomic group. Using the output gene trees from EukPhylo part 2, (see example template Table S11 in Figshare and the manual for more details), clade-based contamination removal examines each node and determines if the set of taxa above that node fits the user-input criteria for selection as a robust clade. This means that the number of target taxa must be above the input threshold, the number of non-target taxa must be below the given threshold, and optionally that the clade includes at least a certain number of 'required' taxa. Users can define a separate set of rules for any number of target taxonomic groups, including a set of 'exceptions,' which are taxa not to remove under any circumstances. After each iteration, sequences belonging to any group of target taxa that do not fall into a selected clade are removed from the tree, which is then rebuilt without those sequences.

Once sequences that fit the input criteria (in both clade-grabbing and sister/subsister mode) have been identified, all sequences belonging to the target taxon that are not in the selected clades are removed from the iteration and written into an output file SequencesRemoved_ContaminationLoop.txt (File S8 in Figshare, a file that can be reused in future analyses). The loop then writes an unaligned amino acid fasta file for each GF without these removed sequences, and runs these through the alignment and the tree-building methods of choice as described above. Using the --n_loops argument the user can specify the maximum number of times this process may repeat. Note that 'Clade Grabbing' likely removes vertically-inherited sequences as well as contaminants and hence should be used with caution.

Case study – Conserved OG analysis

To demonstrate the power of EukPhylo, we provide a case study of analyses of 500 gene families sampled from 1000 species. We downloaded genome and transcriptome data from GenBank and combined them with a subset of our in-house transcriptomic data (Table S1 in Figshare). We used EukPhylo part 1 to produce fasta formatted files containing CDS (genomes) or assembled transcripts (transcriptomes). Starting with more than 2500 species, we made a preliminary taxon list of about 1,200 diverse eukaryote and prokaryote genome and transcriptome assemblies based on the number of sequences assigned to GFs by EukPhylo part 1, and the number of GFs identified in the process. For each sample, we manually

inspected graphs of silent-site GC content (GC3S) vs. effective number of codons for all sequences (output by EukPhylo part 1, File S3 in Figshare) to assign upper and lower bounds of GC3S, and removed sequences outside of these bounds (Table S1 in Figshare). To further reduce the number of taxa in our dataset, we combined this analysis with a phylogeny-informed assessment of contamination in each genome/transcriptome: we selected GFs based on diversity, and chose 800 GFs with the greatest diversity of eukaryotes. We then created MSAs and trees with EukPhylo part two (using five iterations of Guidance). Describing the sister relationship of each taxon in the trees (using the utility script ContaminationBySister.py) allowed us to assess their performance of taxa and to generate a final list of 1,000.

In developing the rules for the 'sisters' mode of the contamination loop, we relied on the output of the utility script ContaminationBySisters.py. We coupled this output with visual-inspection of gene trees colored by minor clades. To set rules for clade-selection mode, we inspected clade sizes in trees to describe clade sizes for well-sampled taxa in the set of trees after the first two runs of the contamination loop in sister/subsister mode (using CladeSizes.py). After final manual curation, we reduced the OG list to 500, based on diversity of taxa, and created a final set of curated trees by running IQ-Tree with model LG+G (File S4 in Figshare).

To test the impact on the species tree of removing GFs with a putative signal of endosymbiotic gene transfer (EGT) we identified, removed putative EGT and then assessed the resulting topologies. We first identified putative primary EGT events with the utility script CladeGrabbing.py, identifying every tree (post ciliate-clade grabbing) that had at least 5 bacteria branching with at least 5 Archaeplastida (Pl_gr, Pl_rh and Pl_gl). To identify putative secondary EGT events, we first ran CladeGrabbing.py to identify all clades containing only photosynthetic taxa across all trees (post ciliate-clade grabbing), and then ran CladeSize.py to help us describe the complexity of these clades. Our goal here was to select GFs with the greatest number and complexity (i.e., number of taxonomic groups per clade) of photosynthetic clades for manual inspection. After manual analysis of the selected trees, we removed a total of 169 GFs from the dataset.

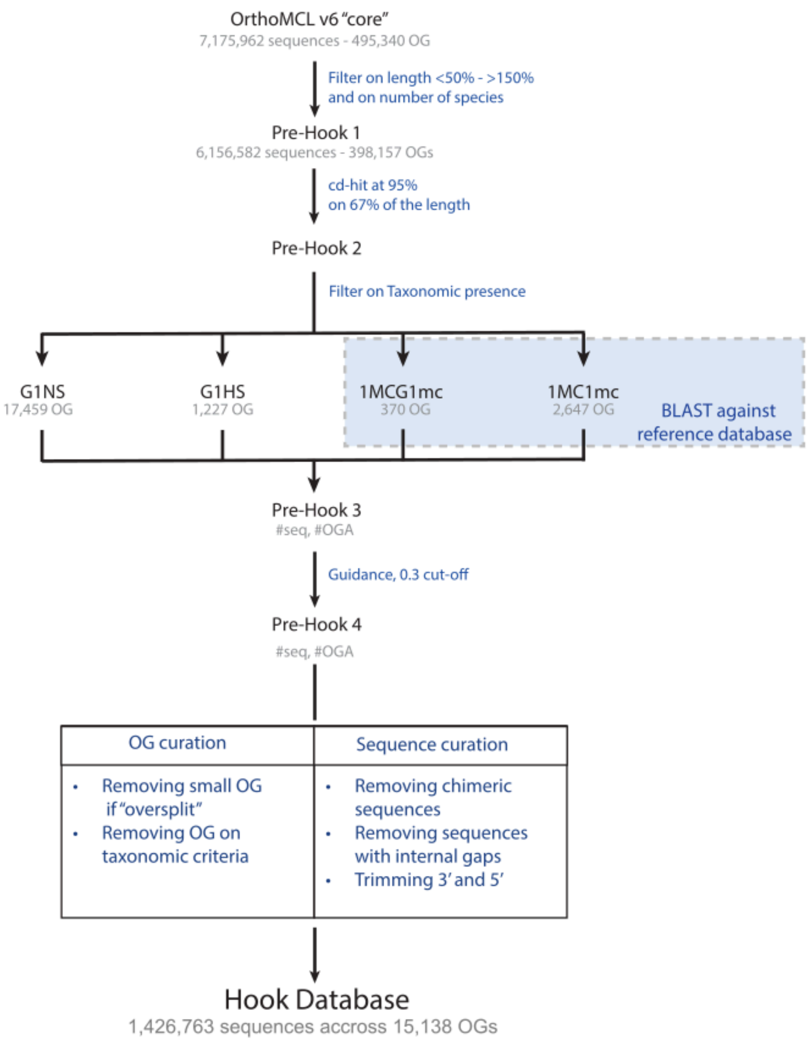

Fig. S1: A description of the development of the Hook Database, used in EukPhylo part 1 for GF assignment (see methods above for more details); the goal here was to develop a set of GFs that would be useful for a variety of studies, but not to capture every eukaryotic GF. Briefly, we started with all sequences from the "core" GFs from OrthoMCL version 6.13, implemented basic length and taxon presence filters ("Pre-Hook 1") and clustered these sequences using CD-Hit ("Pre-Hook 2"). We filtered again on taxonomic presence, curated some GFs by hand to generate "Hook 6.1", and ran each GF through Guidance for homology assessment ("Hook 6.2"). We then curated again to remove GFs that were over-split or with low diversity, and sequences of putatively low quality, to produce the final reference database. (OG = GF = Gene Families)

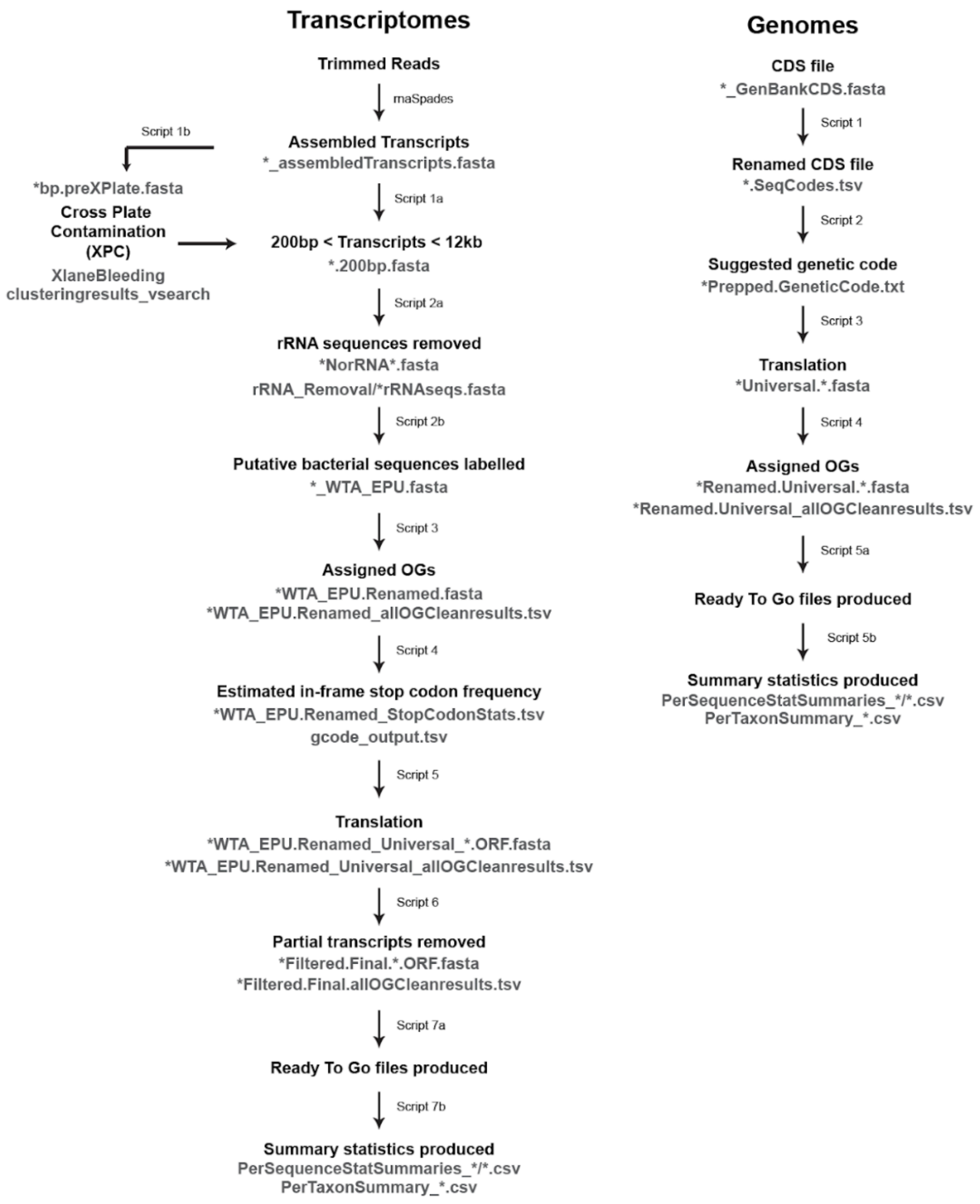

Fig S2: A flowchart describing the steps in EukPhylo part 1, with script numbers corresponding to script names on the GitHub page (https://github.com/Katzlab/EukPhylo-6). See the methods in the Supplemental Text ("Transcriptomic pipeline - an in-depth description") for details.

Figure S3

(a) EndPTL, 50% gap trimmed, Asteroid.

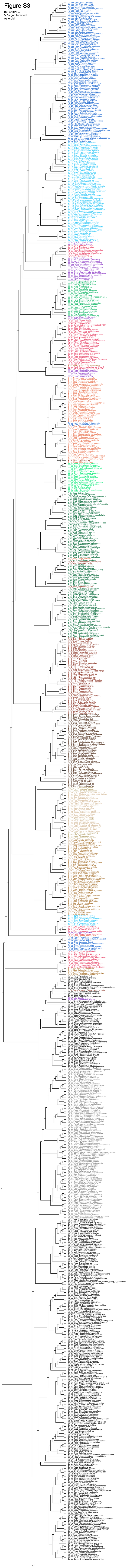

Figure S3 (b)
EndPTL,
50% gap trimmed,
concatenated.

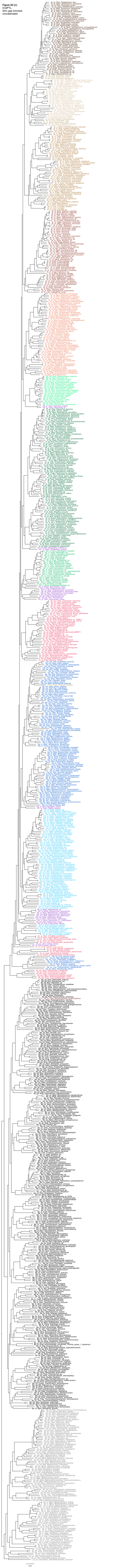

Figure S3 (c) Sisters removed, 50% gap trimmed, Asteroid

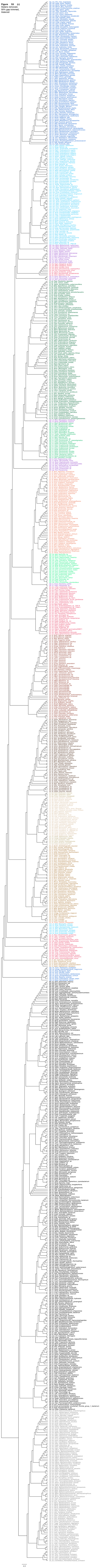

Figure S3 (d) Sisters removed, 50% gap trimmed, concatenated.

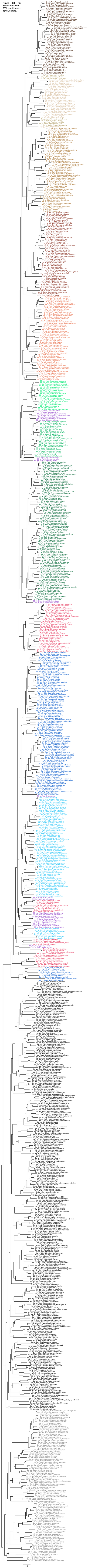

Figure S3 (e) Clade-grabbed, 50% gap trimmed, Asteroid.

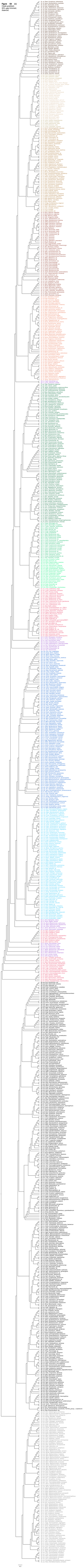

Figure S3 (f) Clade-grabbed, 50% gap trimmed, concatenation

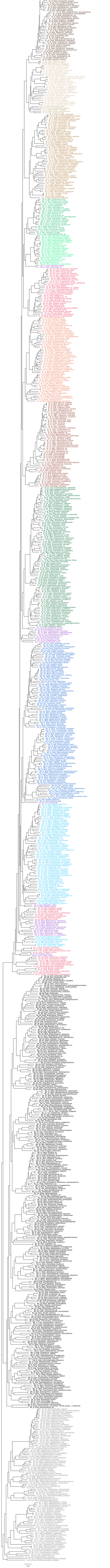

Figure S3 (g)
Putative EGT removed,
50% gap trimmed,
Asteroid

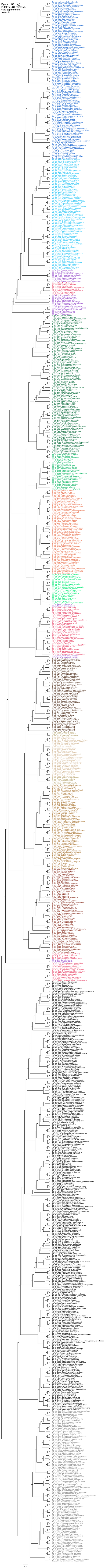

Figure S3 (h)
Putative EGT removed, 50% gap trimmed, concatenated.

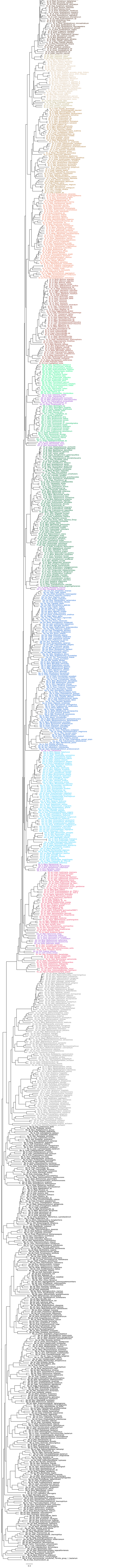

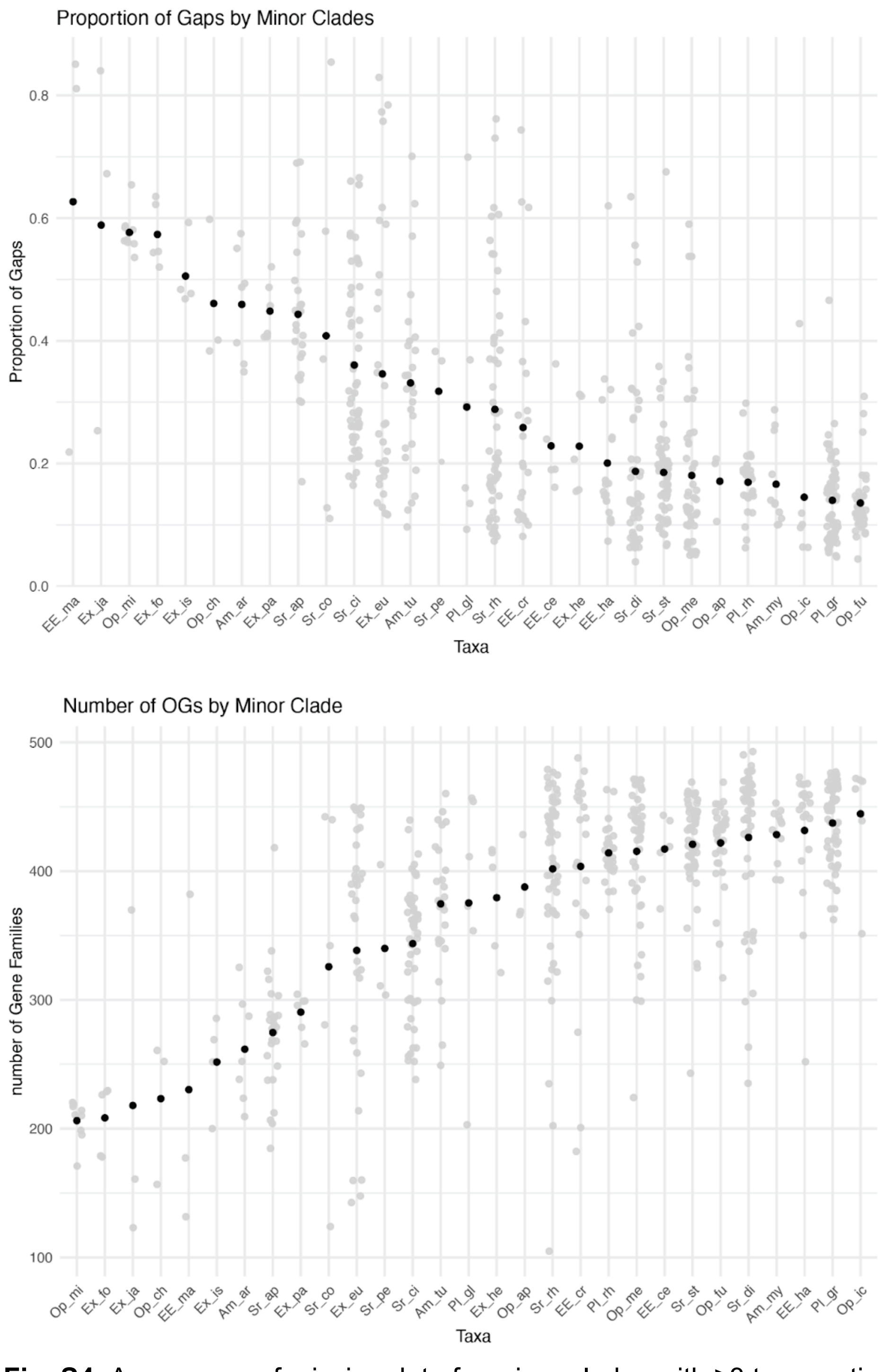

Fig. S4. A summary of missing data for minor clades with ≥3 taxa, estimated after the sister/subsister step in our contamination loop (see Fig. 4). Each grey point represents a species (Table S1 in Figshare), and the black points are mean values. Several of the clades with the greatest proportion of gaps (a) and that are present in the fewest GFs (b), such as microsporidia (Op_mi), Fornicata (Ex_fo), Jakobids (Ex_ja) and Preaxostyla (Ex_is) are those that show the greatest variation in placement in the species tree and those that tend to fall towards the root across analyses (Fig 4a-d).

Supplemental References

Cantalapiedra, C. P., Hernández-Plaza, A., Letunic, I., Bork, P., & Huerta-Cepas, J. (2021). eggNOG-mapper v2: Functional Annotation, Orthology Assignments, and Domain Prediction at the Metagenomic Scale. *Molecular Biology and Evolution*, *38*(12), 5825–5829. https://doi.org/10.1093/molbev/msab293

Costello, M., Fleharty, M., Abreu, J., Farjoun, Y., Ferriera, S., Holmes, L., Granger, B., Green, L., Howd, T., Mason, T., Vicente, G., Dasilva, M., Brodeur, W., DeSmet, T., Dodge, S., Lennon, N. J., & Gabriel, S. (2018). Characterization and remediation of sample index swaps by non-redundant dual indexing on massively parallel sequencing platforms. *BMC Genomics*, *19*(1), 332. https://doi.org/10.1186/s12864-018-4703-0

Huerta-Cepas, J., Szklarczyk, D., Heller, D., Hernández-Plaza, A., Forslund, S. K., Cook, H., Mende, D. R., Letunic, I., Rattei, T., Jensen, L. J., von Mering, C., & Bork, P. (2019). eggNOG 5.0: A hierarchical, functionally and phylogenetically annotated orthology resource based on 5090 organisms and 2502 viruses. *Nucleic Acids Research*, *47*(D1), D309–D314. https://doi.org/10.1093/nar/gky1085

Klopfenstein, D. V., Zhang, L., Pedersen, B. S., Ramírez, F., Warwick Vesztrocy, A., Naldi, A., Mungall, C. J., Yunes, J. M., Botvinnik, O., Weigel, M., Dampier, W., Dessimoz, C., Flick, P., & Tang, H. (2018). GOATOOLS: A Python library for Gene Ontology analyses. *Scientific Reports*, *8*(1), 10872. https://doi.org/10.1038/s41598-018-28948-z

MacConaill, L. E., Burns, R. T., Nag, A., Coleman, H. A., Slevin, M. K., Giorda, K., Light, M., Lai, K., Jarosz, M., McNeill, M. S., Ducar, M. D., Meyerson, M., & Thorner, A. R. (2018). Unique, dual-indexed sequencing adapters with UMIs effectively eliminate index cross-talk and significantly improve sensitivity of massively parallel sequencing. *BMC Genomics*, *19*(1), 30. https://doi.org/10.1186/s12864-017-4428-5

Sela, I., Ashkenazy, H., Katoh, K., & Pupko, T. (2015). GUIDANCE2: Accurate detection of unreliable alignment regions accounting for the uncertainty of multiple parameters. *Nucleic Acids Research*, *43*(W1), W7-14. https://doi.org/10.1093/nar/gkv318
